# Genetic diversity of *Bordetella bronchiseptica* isolates obtained from primates

**DOI:** 10.3389/fmicb.2025.1571660

**Published:** 2025-06-26

**Authors:** Tracy L. Nicholson, Sarah M. Shore, Yihui Wang, Lindsey Zimmerman, Tod J. Merkel

**Affiliations:** ^1^National Animal Disease Center, Agricultural Research Service, USDA, Ames, IA, United States; ^2^Division of Bacterial, Parasitic and Allergenic Products, Center for Biologics Evaluation and Research, FDA, Silver Spring, MD, United States

**Keywords:** *Bordetella bronchiseptica*, whole-genome sequencing (WGS), comparative genomics, virulence factor, antimicrobial resistance (AMR), primate

## Abstract

*Bordetella bronchiseptica* is a highly contagious bacterial respiratory pathogen with a broad host range of wild and domesticated mammals that can cause a variety of clinical disease outcomes ranging from asymptomatic carriage to severe pneumonia. The goal of this study was to evaluate the genetic diversity of *B. bronchiseptica* isolates obtained from primates and evaluate the antimicrobial resistance harbored by these isolates. Two isolates were identified as belonging to *B. bronchiseptica* lineage II and 13 isolates represented new sequence types within *B. bronchiseptica* lineage I clonal complex 6. The lineage II isolates harbored the lowest sequence identity observed across all genes evaluated and did not contain several well characterized virulence and fimbrial genes. Western blotting revealed no reactivity to a lineage II strain when using antibodies generated against pertactin (PRN) from a lineage I-1 strain or antibodies generated against a domain of filamentous haemagglutinin (FHA) from a lineage I-1 strain. Isolates harbored variation within the *wbm* locus containing genes encoding for the expression of antigenically distinct O-antigen types and the *cya* operon was replaced by the *ptp* operon in several isolates, expanding the phylogenetic distribution of this operon replacement. Thirteen isolates exhibited phenotypic resistance to four antibiotic classes tested, however the *Bordetella*-specific β-lactamase was the only antimicrobial resistance gene identified. Collectively, the data in this report expands the known phylogenetic diversity and genetic variation of *B. bronchiseptica* isolates.

## Introduction

*Bordetella bronchiseptica* is a highly contagious respiratory bacterial veterinary pathogen that can cause a variety of clinical disease outcomes ranging from asymptomatic carriage to severe bronchopneumonia (Mattoo and Cherry, 2005; Chambers et al., 2019; Brockmeier et al., 2019). *B. bronchiseptica* is closely related to *Bordetella pertussis* and *Bordetella parapertussis_hu_*, the causative agents of whooping cough or pertussis in humans and combined are collectively referred to as the “classical bordetellae” (Goodnow, 1980; Parkhill et al., 2003; Preston et al., 2004; Diavatopoulos et al., 2005). *B. parapertussis* consists of two genetically distinct lineages: *B. parapertussis_hu_*, which is only known to infect humans, and *B. parapertussis_ov_*, which is only known to infect sheep (Porter et al., 1994). The human-restricted pathogens, *B. pertussis* and *B. parapertussis_hu_*, are considered to have evolved independently from a *B. bronchiseptica* ancestor (Diavatopoulos et al., 2005; Preston et al., 2004; Parkhill et al., 2003). In addition to their close genetic relatedness, classical bordetellae harbor many of the same virulence factors, which are similarly regulated (Linz et al., 2016; Chen and Stibitz, 2019; Parkhill et al., 2003). Despite these similarities, the classical bordetellae differ in traits such as host specificity, disease severity, and duration of infection. In addition to only infecting humans, *B. pertussis* and *B. parapertussis_hu_* also lack an animal reservoir and the ability to survive in the environment (Hewlett et al., 2014; Pinto and Merkel, 2017). In contrast, *B. bronchiseptica* infects a variety of mammals, often establishing chronic infections, and is capable of surviving in the environment (Goodnow, 1980; Brockmeier et al., 2019; Badhai and Das, 2021). While rare, human *B. bronchiseptica* infections have been reported (Barcala Salido et al., 2022; Register et al., 2012; Agarwal et al., 2022; Moore et al., 2022; Woolfrey and Moody, 1991).

A key regulatory mechanism that is shared among the classical bordetellae is that the majority of virulence gene expression is regulated by a two-component sensory transduction system encoded by the *bvg* locus (Chen and Stibitz, 2019; Cotter and Jones, 2003). This locus contains BvgS, a histidine kinase sensor protein, and BvgA, a DNA-binding response-regulator protein. In response to a variety of environmental cues, BvgAS controls the expression of phenotypic phases transitioning between a virulent (Bvg^+^) phase and a non-virulent (Bvg^−^) mode (Chen and Stibitz, 2019; Cotter and Jones, 2003; Nicholson et al., 2012). During the virulent Bvg^+^ phase, the BvgAS system is fully active and virulence-activated genes (vags), such as filamentous hemagglutinin (FHA), pertactin (PRN), fimbriae, dermonecrotic toxin (DNT), adenylate cyclase toxin (ACT), and a type III secretion system (T3SS), are fully expressed (Cotter and Jones, 2003; Nicholson et al., 2012; Nicholson, 2007; Nicholson et al., 2024).

The overwhelming majority of publicly available genomic sequencing data for the classical bordetellae is derived from clinical *B. pertussis* isolates due to the medical importance of whooping cough and the epidemiological surveillance of vaccine-driven selection occurring among *B. pertussis* isolates. The goals of the current study were to fill this gap by utilizing whole-genome sequencing (WGS) analysis to evaluate the genetic diversity of *B. bronchiseptica* (Bb) isolates obtained from non-human primates and assess the phenotypic and genotypic antimicrobial resistance (AMR) of these isolates. Collectively, the data in this report broadens the known phylogenetic diversity and genetic variation of Bb isolates.

## Materials and methods

### Isolate information

Five Bb isolates were obtained from nasopharyngeal samples collected from baboons and 14 Bb isolates were obtained from nasopharyngeal samples collected from rhesus monkeys (Supplementary Table 1). All isolates were obtained during routine screening for various pathogens, while animals were housed in large groups. Isolates M2020-2 and M2020-4 were obtained from baboons housed together and these are the only isolates obtained from animals known to be housed together. All isolates were either obtained from samples collected as part of previous studies or were obtained from samples submitted as part of field case investigations and did not require Institutional Animal Care and Use Committee (IACUC) approval. Frozen stocks (−80°C in 30% glycerol) of Bb isolates were received and then streaked onto tryptic soy agar containing 5% sheep blood (Becton, Dickinson and Co. Franklin Lakes, NJ) and incubated overnight at 37°C with 5% CO_2_. Classical bordetellae isolates used in the Bridel et al. (2022) study and previously characterized Bb strains RB50 and KM22, TN27 (KM22 *ΔfhaB*), and TN28 (KM22 *Δprn*) were also included in the analyses (Supplementary Table 1) (Parkhill et al., 2003; Nicholson et al., 2020; Nicholson et al., 2009).

### Whole-genome sequencing, assembly, and annotation

WGS data was acquired from a variety of methods. For genomic DNA extraction from all isolates except M2020-2 and M2020-4, single colonies were inoculated into lysogeny broth (LB) and grown aerobically at 37°C overnight in a shaking incubator (250 rpm). The High Pure PCR Template Preparation Kit (Roche Diagnostics Corp., Indianapolis, IN) was used to purify total genomic DNA. The Qubit 1X dsDNA BR Assay Kit (Life Technologies, Eugene, OR) was used to assess quality standards and determine DNA concentration. WGS assemblies for isolates M2020-3, M2020-5, C2020-1, C2020-2, C2020-3, C2020-4, C2020-5, C2020-9, C2020-10, C2020-11, C2020-12, C2020-13, C2020-14, and C2020-15 were obtained using Illumina short read data. Library preparation was performed using a custom Illumina TruSeq-style protocol (Arbor Biosciences, Ann Arbor, MI) and sequenced on an Illumina NovaSeq 6000 in 150 bp paired-end mode. The Illumina datasets were assessed for quality using FastQC^1^ and adapter trimming performed using BBduk.^2^ Assembly was performed using SPAdes v. 3.15.4 in --careful mode (Bankevich et al., 2012). Resulting assemblies were filtered to retain only contigs greater or equal to 1,500 bases in length.

For isolates M2020-2 and M2020-4, single colonies were inoculated into LB broth and grown overnight aerobically at 37°C. Bacterial cells were centrifuged at 5,000 rpm to pellet, washed in 10 mL PBS and pelleted again. The cell pellets were frozen overnight at-20°C. The pellets were resuspended in 2 mL of 25% sucrose in TE (10 mM Tris, 1 mM EDTA, pH 8.0). Fifty microliters of 100 mg/mL lysozyme (Roche, Indianapolis, IN) was added and the samples incubated for 1 h at 37°C. Next, 100 μL of 20 mg/mL Proteinase K (Roche, Indianapolis, IN), 30 μL RNase A (Roche, Indianapolis, IN), 400 μL 0.5 M EDTA (pH 8.0), and 250 μL of 10% sarkosyl were added to the samples, which were then incubated on ice for 2 h, followed by overnight incubation in a 50°C water bath. Sample volume was adjusted to 7.5 mL with TE and mixed with 5 mL phenol:chloroform:isoamyl alcohol (25:24:1), then centrifuged for 15 min at 4,000 rpm to separate the phases. The aqueous phase was transferred to a fresh tube and the phenol:chloroform:isoamyl alcohol phase separation was repeated twice. The aqueous phase was transferred to a new tube and mixed with an equal volume of chloroform:isoamyl alcohol (24:1), followed by centrifugation for 5 min at 4,000 rpm to separate the phases. The chloroform:isoamyl alcohol extraction was repeated one additional time. To the final aqueous phase, 2.5 volumes 100% ethanol was added and incubated at −20°C for 15 min to precipitate the DNA, followed by centrifugation at 4,000 rpm for 10 min. The pellet was washed with 10 mL cold 70% ethanol, air dried and resuspended in 250 μL TE. DNA concentrations were determined by UV spectrophotometry and assed for quality standards.

Complete genome assemblies for isolates M2020-2 and M2020-4 were obtained using a hybrid assembly approach combining Illumina short sequencing reads and PacBio long reads. For long read PacBio sequencing, library preparation was performed using the SMRTbell Express Template Prep Kit 2.0 (PacBio, Menlo Park, CA) and sequenced on a Sequel II system using an 8 M SMRT Cell. For short read Illumina sequencing, libraries were prepared using the KAPA Hyper Prep Kit (Roche Sequencing Solutions, Pleasanton, CA) and sequenced on an Illumina NovaSeq 6000 instrument in 150 bp paired-end mode. The Illumina data was assessed for quality using FastQC (see text footnote 1) and adapter trimming performed using BBduk (see text footnote 2). Assembly of the long read data was performed using Canu v. 2.2 (Koren et al., 2017) with default settings. Circlator v. 1.50 (Hunt et al., 2015) was used to generate a closed circular assembly with no overlaps and rotate the sequence to start at the *dnaA* gene. Pilon v. 1.23 (Walker et al., 2014) was used to polish the assembly using the Illumina short read data. Multiple rounds of Pilon polishing were performed, until no additional corrections were made.

Complete genome assembly of isolate C2020-8 was performed using a hybrid approach using a combination of long and short reads. Short read Illumina data was obtained using the same library preparation and sequencing as described above for the short-read only assemblies. For long-read PacBio sequencing, a HiFi Multiplex library with 10–20 kb insert size was prepared and sequenced on a Sequel II system using a SMRT Cell 8 M in CCS/HiFi mode. Long read data was assembled using Flye v. 2.9.1 with options --pacbio-hifi mode --asm-coverage 100-g 5.3 m (Kolmogorov et al., 2019), resulting in a closed, circular assembly. The Illumina data was then used to polish the assembly using Pilon v. 1.23 (Walker et al., 2014). The final assembly was rotated to start at the *dnaA* gene.

Isolates M2020-1 and C2020-7 were assembled using a hybrid approach combining Illumina short read data and Nanopore long read data. Short read Illumina data was obtained using the same library preparation and sequencing as described above for the short-read only assemblies. The genomic library for Nanopore sequencing was prepared with the SQK-RBK004 Rapid Barcoding Kit (Oxford Nanopore, Oxford, United Kingdom), following the manufacturer’s instructions. Sequencing was performed using a MinION Mk1C instrument with a MIN106D flow cell (version R9). The run length was 72 h and base calling was performed using Guppy v. 6.2.11 (high-accuracy mode, minimum read length of 200 bp, minimum Q score of 9).

Long read data was assembled using Flye v. 2.9.1 with settings --nano-raw-g 5.3 m (Kolmogorov et al., 2019) followed by error-correction with Medaka v. 1.11.1 (Oxford Nanopore, Oxford, United Kingdom). For both isolates, this resulted in assembly of a single, closed circular chromosome. The Illumina short read data was then used to polish the long read assemblies using Polypolish v. 0.5.0 and POLCA v. 4.1.0 as described in Wick et al. (2017) and Zimin and Salzberg (2020). The resulting assemblies were rotated to start at the *dnaA* gene.

Unless otherwise specified, default software settings were used. All assemblies were annotated using the NCBI Prokaryotic Genome Annotation Pipeline (PGAP) v. 6.5 (all assemblies except C2020-7 and M2020-1) or v. 6.7 (C2020-7 and M2020-1) (Tatusova et al., 2016).

### Comparative genomic analysis

Multi-locus sequence typing (MLST) was performed *in silico* utilizing the BIGSdb-Pasteur databases hosted by the Institut Pasteur.^3^ Average nucleotide identity (ANI) for analyzed genomes was calculated using FastANI, which uses an algorithm based on MinHash mapping to calculate pairwise genome-to-genome ANI values (Jain et al., 2018). In R, the ComplexHeatmap package (Gu et al., 2016) was used to generate a distance matrix heatmap clustered by hierarchical clustering using a complete linkage method with Euclidean distance.

The presence of the 9 bp deletion found in the complete genome assembly of M2020-4 *bvgS* compared to M2020-2 was verified by Sanger sequencing. Briefly, the *bvgS* gene was PCR amplified from 2 independent genomic DNA samples from each isolate using primers bvgSq1-for (5′-TGGTGGAACTGATAGACCTCGCCAAACGCAACAAT-3′) and bvgSq1-rev (5′-TACCGCCGCGAGATGCCGCCCAATTATCCGTAAGG-3′). PCR was performed using Platinum SuperFi II PCR Master Mix (ThermoFisher, Waltham, MA) with manufacturer’s recommended conditions. The PCR product was purified using the Wizard SV Gel and PCR Clean-Up System (Promega, Madison, WI) and sequenced using primers Sfor-3F (5′-ATCAGCGCCAGCTACTAC-3′) and SMS-5R (5′-GAGCTGCACGACGCCAAG-3′).

A custom database comprised of known *Bordetella* virulence-associated genes in Bb strain KM22 was used to identify corresponding homologues by BLASTN (Altschul et al., 1997) searches and the percent identity for each gene relative to the reference gene from Bb strain KM22 (accession # CP022962) was determined. Genes and locus tag information is provided in Supplementary Table 2.

Non-redundant or unique amino acid sequences of either FHA or PRN from analyzed *Bordetella* isolates containing a full-length nucleotide gene sequence were aligned using MAFFT (Katoh and Standley, 2013). Amino acid sequences were evaluated using hierarchical clustering based on the pairwise percent identity between isolates. Clustering was performed in R using the ComplexHeatmap package, with complete linkage and Euclidean distance as the clustering method (Gu et al., 2016). The amino acid sequences were additionally used to infer approximately-maximum-likelihood phylogenetic trees with FastTree (v2.11) and iTOL (Price et al., 2010; Letunic and Bork, 2024).

O-antigen loci were identified by a BLASTN (Altschul et al., 1997) search for the genes known to flank the O-antigen locus, using the RB50 genes BB0120 and *wlbL*/BB0145 (Vinogradov et al., 2010) as the query sequences. Sequences were compared to the known O-antigen Bb serotypes O1, O2, and O3 and the *B. parapertussis* Bpp5 O-antigen locus (Vinogradov et al., 2010; Hester et al., 2013; Hester et al., 2015) by MAFFT alignment (Katoh and Standley, 2013).

To determine the presence of the *cya* or *ptp* loci, *in silico* PCR was performed in Geneious Prime 2023.0.1^4^ using the primer sets described in Sukumar et al. (2014) to identify the respective operons and further analyze genomic regions encoding these loci.

Comparison of genes encoding fimbrial protein subunits within the *fimNX* region was performed by BLASTN search (Altschul et al., 1997) to identify the region and MAFFT alignment (Katoh and Standley, 2013) to categorize the gene families.

### Phenotypic and genomic AMR analysis

Phenotypic antibiotic resistance was determined using the broth microdilution method by National Veterinary Services Laboratories (Ames, IA) following standard operating procedures. Minimum inhibitory concentrations (MICs) were determined for each isolate using the Trek BOPO7F and GNX3F plates (Thermo Fisher Scientific Inc., Oakwood Village, OH) with *Escherichia coli* ATCC 25922 (ATCC, Manassas, VA) serving as the quality control strain. MICs were evaluated in accordance with Clinical Laboratory Standards Institute (CLSI) recommendations based on the VET01S and M100Ed34 standards for resistance interpretations after incubation for 24 h (CLSI, 2024a; CLSI, 2024b; Pruller et al., 2015).

Result interpretation breakpoints used for Bb were values provided by the CLSI guidelines for ampicillin, clindamycin, florfenicol, tildipirosin, tulathromycin (CLSI, 2024a). Since breakpoints specific to Bb are limited, breakpoints used for treating *Pseudomonas aeruginosa* infections were used for amikacin, gentamicin, aztreonam, doripenem, imipenem, meropenem, piperacillin/tazobactam, ticarcillin/clavulanic acid, cefepime, cefotaxime, ceftazidime, ciprofloxacin, levofloxacin, colistin, polymixin B, doxycycline, minocycline, tetracycline, tigecycline (CLSI, 2024a; CLSI, 2024b). Breakpoints used for treating *Pasteurella multocida* infections were used for neomycin, spectinomycin, penicillin, ceftiofur, danofloxacin, enrofloxacin, gamithromycin, tiamulin (CLSI, 2024a). Breakpoints used for treating *Mannheimia haemolytica* infections were used for tilmicosin (CLSI, 2024a). Isolates were considered resistant to sulfadimethoxine when MIC was equal to or exceeded 256 μg/mL, and isolates were considered resistant to trimethoprim/sulfamethoxazole when MIC exceeded 2 μg/mL (Vilaro et al., 2023). Antimicrobial susceptibility data (AST), along with test rages and clinical breakpoints used for interpretations, for all isolates are listed in Supplementary Table 3.

An *in silico* search for antimicrobial resistance (AMR) genes was performed using AMRFinderPlus v. 3.12.8 with database version 2024-05-02.2 (Feldgarden et al., 2019). The input option --nucleotide was used to analyze the assembled genome FASTA sequences with default settings. The database used the *blaBOR* protein sequence from *B. bronchiseptica* RB50 as the reference for the search (accession WP_010926363.1). The RB50 *blaBOR* nucleotide sequence (locus_tag BB2049) was then employed to compare to the *blaBOR* gene sequences identified in the Bb primate isolates.

### Western blots

Selected isolates were grown on Bordet-Gengou (BG) agar (Difco, Sparks, MD) + streptomycin (40 μg/μL) plates, and a single colony was inoculated into 3 mL Stainer-Scholte broth + 40 μg/μL streptomycin (SS/Sm) and grown aerobically overnight at 37°C. This overnight culture was then used to inoculate 10 mL SS/Sm broth to an OD600 of 0.05, which was grown aerobically overnight at 37°C. The cultures were harvested by centrifugation, and the cell pellets were resuspended in 1/5th volume 8 M urea. The lysates were heated to 95°C for 10 min, then cooled to room temperature. The Qubit Protein BR Kit (Thermo Fisher, Rockford, IL) was used to determine protein concentration and the lysates stored at −80°C prior to use, the lysates were diluted in 4X LDS Sample Buffer (Life Technologies, Carlsbad, CA) to 500 ng/μL or 250 ng/μL in 1X LDS + 4 M urea. SDS-PAGE was performed using NuPAGE 7% Tris-Acetate gels (Life Technologies, Carlsbad, CA) loaded and run under denaturing conditions according to the manufacturer’s recommendations. Transfer to PVDF membranes was performed using an Xcell Blot Module (Life Technologies, Carlsbad, CA) following manufacturer’s instructions. Membranes were blocked for 1 h at room temperature in 1X PBS + 0.1% Tween-20 + 5% non-fat dry milk. Primary antibody incubations were performed overnight at 4°C in 1X PBS + 0.1% Tween-20 + 5% non-fat dry milk. Membranes were washed 4 × 5 min in 1X PBS + 0.1% Tween-20. Secondary antibody incubations were for 2 h at room temperature in 1X PBS + 0.1% Tween-20 + 2.5% non-fat dry milk. Membranes were washed 4 × 5 min in 1X PBS + 0.1% Tween-20 and then incubated with SuperSignal West Pico PLUS Chemiluminescent Substrate (Life Technologies, Carlsbad, CA) per manufacturer’s protocol. Digital images were obtained using an Azure 600 imager (Azure Biosystems, Dublin, CA). KM22 FHA residues 400–1,025 were used to generate mouse polyclonal α-FHA-heparin binding domain antibodies and KM22 FHA residues 2,000–2,525 were used to generate mouse polyclonal α-FHA-mature C-terminal domain antibodies (Genscript United States, Inc., Piscataway, NJ). Primary antibodies to FHA were used at 1:500 dilutions and detected with the secondary antibody goat anti-mouse IgG-HRP 31432 (Life Technologies, Carlsbad, CA) used at a 1:10,000 dilution. The primary antibody to pertactin, α-PRN 29854, is a rabbit polyclonal antibody generated against purified PRN from a Bb swine isolate produced at the NADC and was used at a 1:5,000 dilution and detected with the secondary antibody goat anti-rabbit IgG-HRP 4030-05 (Southern Biotech, Birmingham, AL).

### Data availability

The genome assemblies have been deposited at DDBJ/ENA/GenBank under the following BioProject accessions: PRJNA954421, PRJNA954729, PRJNA954420, and PRJNA954735. The sequencing data has been deposited in the Sequence Read Archive (SRA) under the following accession numbers: SRP435095, SRP435093, SRP435092, and SRP435108. Detailed information regarding BioSample, GenBank, and SRA accession numbers is provided in Supplementary Table 1.

## Results

### Phylogenetic analysis of Bb primate isolates

MLST was performed to begin characterizing the Bb primate isolates. One new *fumC* allele was identified in baboon isolates M2020-1, M2020-5, and M2020-3 and one new *tyrB* allele was identified in all rhesus monkey isolates (C2020-1, C2020-2, C2020-3, C2020-4, C2020-5, C2020-8, C2020-9, C2020-10, C2020-11, C2020-12, C2020-13, C2020-14, and C2020-15) except for C2020-7. Baboon isolates M2020-2 and M2020-4 were determined to be ST67 and M2020-1, M2020-5, and M2020-3 were assigned to ST111, a new ST. Isolate C2020-7 was determined to be ST33, while all other rhesus monkey isolates were assigned to ST109, a new ST (Table 1).

**Table 1:** Isolate information, MLST allele, sequence type (ST), and lineage designation.

Isolate name	Host	Assembly	*adk* ^a^	*fumC*	*glyA*	*tyrB*	*icd*	*pepA*	*pgm*	ST^a^	Lineage^c^
M2020-4	Baboon	Closed	5	3	13	6	10	3	10	67	II
M2020-2	Baboon	Closed	5	3	13	6	10	3	10	67	II
M2020-1	Baboon	Closed	2	**16**^b^	4	1	1	7	2	**111**	I-1
M2020-5	Baboon	Draft	2	**16**	4	1	1	7	2	**111**	I-1
M2020-3	Baboon	Draft	2	**16**	4	1	1	7	2	**111**	I-1
C2020-8	Rhesus monkey	Closed	2	2	4	**15**	1	4	2	**109**	I-1
C2020-1	Rhesus monkey	Draft	2	2	4	**15**	1	4	2	**109**	I-1
C2020-2	Rhesus monkey	Draft	2	2	4	**15**	1	4	2	**109**	I-1
C2020-3	Rhesus monkey	Draft	2	2	4	**15**	1	4	2	**109**	I-1
C2020-4	Rhesus monkey	Draft	2	2	4	**15**	1	4	2	**109**	I-1
C2020-5	Rhesus monkey	Draft	2	2	4	**15**	1	4	2	**109**	I-1
C2020-9	Rhesus monkey	Draft	2	2	4	**15**	1	4	2	**109**	I-1
C2020-10	Rhesus monkey	Draft	2	2	4	**15**	1	4	2	**109**	I-1
C2020-11	Rhesus monkey	Draft	2	2	4	**15**	1	4	2	**109**	I-1
C2020-12	Rhesus monkey	Draft	2	2	4	**15**	1	4	2	**109**	I-1
C2020-13	Rhesus monkey	Draft	2	2	4	**15**	1	4	2	**109**	I-1
C2020-14	Rhesus monkey	Draft	2	2	4	**15**	1	4	2	**109**	I-1
C2020-15	Rhesus monkey	Draft	2	2	4	**15**	1	4	2	**109**	I-1
C2020-7	Rhesus monkey	Closed	2	2	4	1	1	7	2	33	I-1

a MLST allele and sequence type (ST) designation determined by BIGSdb-Pasteur database hosted by the Institut Pasteur (https://bigsdb.pasteur.fr).

b New alleles and STs designated by bold type.

c Lineage determination based on ST and confirmed by hierarchical clustering of ANI values for the *B. bronchiseptica* primate isolates and the classical bordetellae isolates used in the Bridel et al. (2022) study.

**Figure 1:**
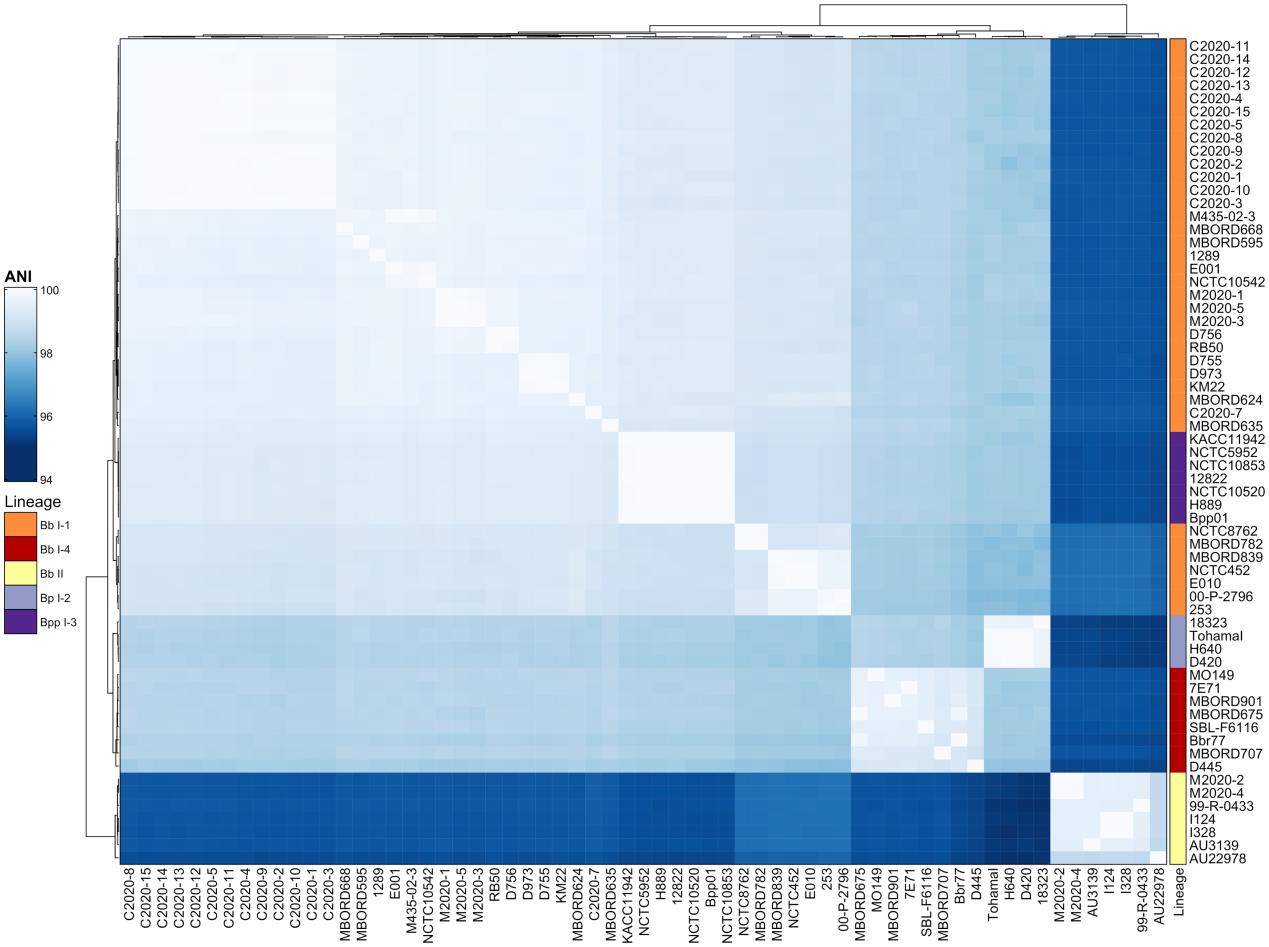
Hierarchically clustered heatmap displaying the relatedness of *Bordetella* isolates based on ANI. Pairwise ANI values for analyzed genomes were used to generate a distance matrix heatmap clustered by hierarchical clustering using a complete linkage method with Euclidean distance. Isolate names are provided at the bottom and right side of heatmap. ANI is represented using the color scale legend shown at the left of the heatmap. Dendrograms are on the left and top sides of the heat map. Lineage designations *B. bronchiseptica* (Bb I-1, Bb I-4, Bb II), *B. parapertussis* (Bpp I-3), and *B. pertussis* (Bp I-2) are shown alongside the isolate names on the right side of the heatmap colored according to the legend shown at the left of the heatmap.

Based on the phylogenetic tree from the cgMLST-based typing method developed by Bridel et al. (2022), baboon isolates M2020-2 and M2020-4 would group into lineage II since both were determined to be ST67, while all other isolates would group into lineage I-1 since they were ST33, ST109, and ST111 (Table 1). To further confirm the lineage for the Bb primate isolates, average nucleotide identity (ANI) values were determined for the Bb primate isolates and the classical bordetellae isolates used in the Bridel et al. (2022) study. Hierarchical clustering of ANI values demonstrated that isolates M2020-1, M2020-5, M2020-3, C2020-1, C2020-2, C2020-3, C2020-4, C2020-5, C2020-8, C2020-9, C2020-10, C2020-11, C2020-12, C2020-13, C2020-14, and C2020-15 were lineage I-1 isolates and isolates M2020-2 and M2020-4 were linage II isolates (Figure 1).

The genome sequences for M2020-2 and M2020-4 were almost identical. The genome sequence assembly for M2020-2 is only 6 bp larger than M2020-4. The differences between the final genome assemblies for these isolates include several nucleotide differences in intergenic regions located in polynucleotide tracts upstream of *fim2*, *fimN*, *bipA*, and *fim3*, as well as a 9-bp deletion in *bvgS* for M2020-4. The deletion in the *bvgS* for M2020-4 corresponds to bps 2,270–2,278 in the *bvgS* sequence for M2020-2 and results in amino acids 758, 759, and 760 missing in BvgS for M2020-4. M2020-4 displays a Bvg-colony morphology, which is likely the result of the mutations in BvgS.

### Comparative analysis of genes encoding BvgAS, FHA, and PRN

While Bb strain RB50 has routinely been used as a reference strain in Bb studies, KM22 was chosen as the reference strain in this study because it shares numerous traits and characteristics that are more consistent with the vast majority of Bb isolates for which genomic data is available in GenBank. KM22 is a ST7 and ST7 is the most common Bb ST in GenBank, whereas RB50 is ST12. Similar to other Bb isolates, KM22 contains functional genes for *tcfA*, *brkA*, *bapC*, and *putative-adhesin 2*, while genes *tcfA*, *brkA*, and *bapC* are pseudogenes in RB50 and *putative-adhesin 2* is absent from the RB50 genome. Gene content at the *fimNX* locus also differs between KM22 and RB50, with KM22 having four fimbrial subunit genes in the locus and RB50 having three genes. The three gene arrangement is uncommon among Bb isolates. Additionally, similar to the majority of Bb isolates, KM22 harbors genes encoding for an O-antigen serotype O2, while RB50 harbors genes encoding for an O-antigen serotype O1.

To examine genomic differences that may influence how the Bb primate isolates interact with their hosts and environment, we compared the nucleotide sequences of genes encoding well-characterized regulatory and virulence factors. Lineage II baboon isolates M2020-2 and M2020-4 harbored the lowest sequence identity for all regulatory and virulence-associated genes evaluated (Table 2; Supplementary Table 2). The nucleotide sequence identity for *bvgA* ranged from 95.08 to 100% and for *bvgS* the identity ranged from 90.25 to 99.97% (Table 2).

**Table 2:** Virulence-associated genes.

Isolate (lineage)	ST^a^	*bvgA* ^b^	*bvgS*	*bvgR*	*fhaB*	*prn*	T3SS *bopB*	T3SS *bopD*	T3SS bopN	T3SS *bsp22*	*bteA*	*dnt*	T6SS *vasA*	T6SS *clpV*
M2020-4 (II)	67	95.08	90.25	88.93	85.55	66.82	90.27	90.87	93.08	93.85	95.7	NP^c^	NP	NP
M2020-2 (II)	67	95.08	90.49	88.93	85.55	66.82	90.27	90.87	93.08	93.85	95.7	NP	NP	NP
M2020-1 (I)	111	100	99.76	100	95.54	99.38	100	99.89	99.91	99.84	99.9	99.75	99.63	99.89
M2020-5 (I)	111	100	99.76	100	95.54	99.38	100	99.89	99.91	99.84	99.9	99.75	99.63	INC^d^
M2020-3 (I)	111	100	99.76	100	95.54	99.38	100	99.89	99.91	99.84	99.9	99.75	99.63	INC
C2020-8 (I)	109	100	99.97	100	94.67	98.87	99.92	100	99.73	100	99.95	99.82	100	99.92
C2020-1 (I)	109	100	99.97	100	94.67	98.87	99.92	100	99.73	100	99.95	99.82	100	INC
C2020-2 (I)	109	99.84	99.97	100	94.67	98.87	99.92	100	99.73	100	99.95	99.82	100	INC
C2020-3 (I)	109	100	99.97	100	94.67	98.87	99.92	100	99.73	100	99.95	99.82	100	INC
C2020-4 (I)	109	100	99.97	100	94.67	98.87	99.92	100	99.73	100	99.95	99.82	100	INC
C2020-5 (I)	109	100	99.97	100	94.67	98.87	99.92	100	99.73	100	99.95	99.82	100	INC
C2020-9 (I)	109	100	99.97	100	94.67	98.87	99.92	100	99.73	100	99.95	99.82	100	INC
C2020-10 (I)	109	100	99.97	100	94.67	98.87	99.92	100	99.73	100	99.95	99.82	100	INC
C2020-11 (I)	109	100	99.97	100	94.67	98.87	99.92	100	99.73	100	99.95	99.82	100	INC
C2020-12 (I)	109	100	99.97	100	94.67	98.87	99.92	100	99.73	100	99.95	99.82	100	99.89
C2020-13 (I)	109	100	99.97	100	94.67	98.87	99.92	100	99.73	100	99.95	99.82	100	INC
C2020-14 (I)	109	100	99.97	100	94.67	98.87	99.92	100	99.73	100	99.95	99.82	100	INC
C2020-15 (I)	109	100	99.97	100	94.67	98.87	99.92	100	99.73	100	99.95	99.82	100	INC
C2020-7 (I)	33	100	99.81	99.89	94.58	99.56	100	99.79	99.73	100	99.95	NP	99.79	99.96
RB50 (I)	12	99.84	99.68	99.89	95.43	98.59	100	99.89	99.91	100	99.95	99.73	99.90	99.96
KM22 (I)	7	100	100	100	100	100	100	100	100	100	100	100	100	100

a Sequence type (ST) designation determined by BIGSdb-Pasteur database hosted by the Institut Pasteur (https://bigsdb.pasteur.fr).

b Percent identity for each gene was determined for each isolate relative to the KM22 orthologue.

c Gene not present (NP) in genome sequence.

d Gene sequence was incomplete (INC) due to location at end of contig resulting in a truncated coding sequence.

The nucleotide sequence identity relative to KM22 for *fhaB*, which encodes for FHA, ranged from 85.55 to 95.54% (Table 2). Primate isolates with the same ST harbored identical *fhaB* gene sequences. The *fhaB* nucleotide sequence from isolate C2020-7 (ST33) was slightly different compared to the other rhesus monkey isolates (Table 2).

**Figure 2:**
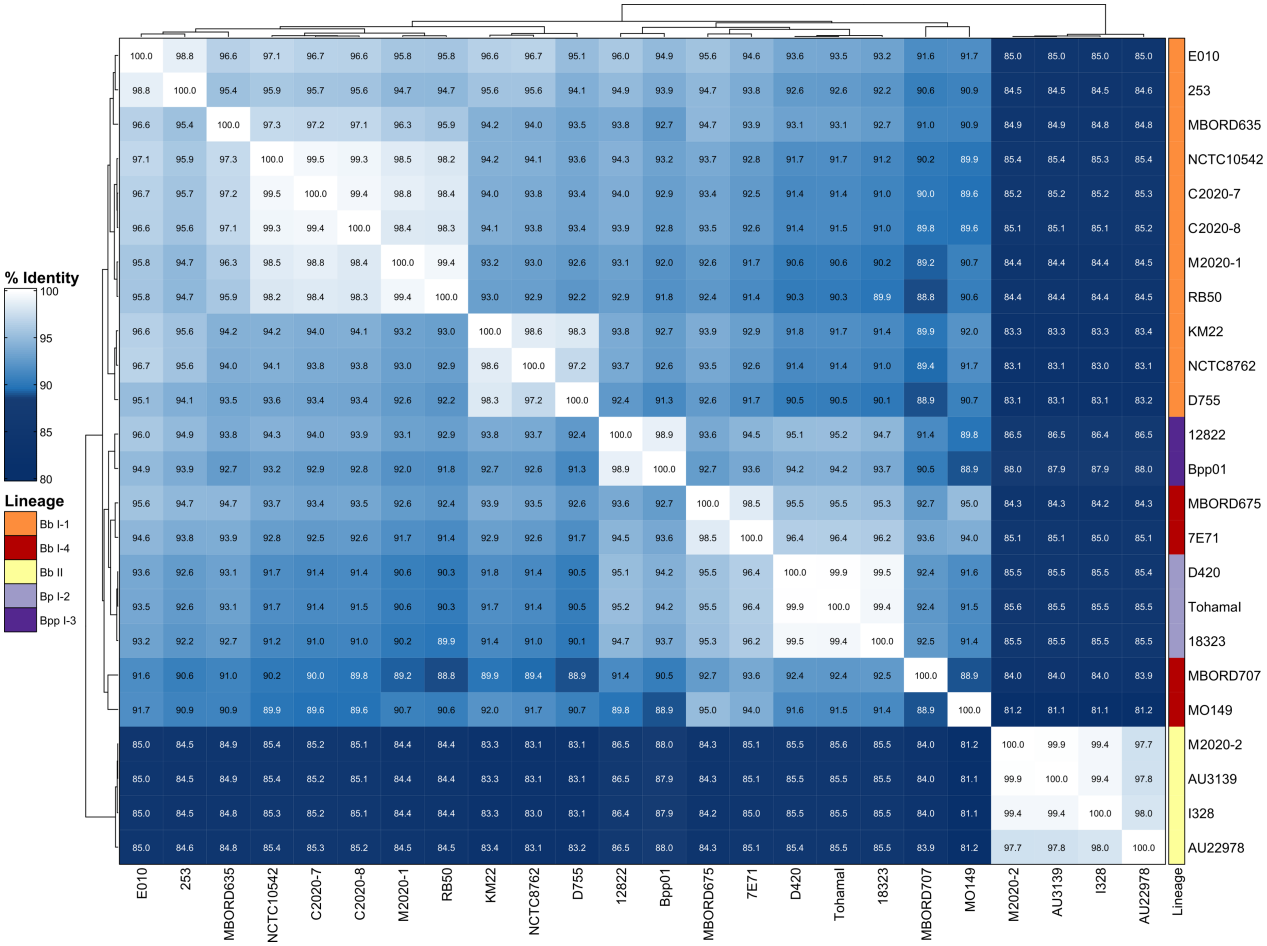
Hierarchically clustered heatmap displaying the relatedness of *Bordetella* isolates based on FHA AA identity. Pairwise AA percent identity of unique FHA sequences from isolate genomes containing a full-length *fhaB* gene was determined and used to generate a distance matrix heatmap clustered by hierarchical clustering using a complete linkage method with Euclidean distance. Isolate names are provided at the bottom and right side of heatmap. FHA AA identity is represented using the color scale shown left of the heatmap. Dendrograms are on the left and top sides of the heat map. Lineage designations *B. bronchiseptica* (Bb I-1, Bb I-4, Bb II), *B. parapertussis* (Bpp I-3), and *B. pertussis* (Bp I-2) are shown alongside the isolate names on the right side of the heatmap colored according to the legend shown left of the heatmap.

To further examine the diversity of FHA, sequence alignments were used to determine pairwise amino acid (AA) identity for FHA sequences from the primate isolates as well as the classical bordetellae isolates used in the Bridel et al. (2022) study. The AA identity was then used to generate a distance matrix heatmap clustered by hierarchical clustering, which revealed a correlation between FHA AA identity and bordetellae lineage (Figure 2). Specifically, the *B. parapertussis* (Bpp) lineage I-3 isolates cluster together, the *B. pertussis* (Bp) lineage I-2 isolates cluster together, and the Bb lineage I-1 isolates cluster together. Two Bb lineage I-4 isolates cluster together and between Bpp lineage I-3 isolates and Bp lineage I-2 isolates, while the other two Bb I-4 isolates cluster together and between and Bb lineage II isolates and Bb I-1 isolates. All Bb lineage II isolates, including baboon isolates M2020-2 and M2020-4, cluster together. The FHA AA identity is conserved among the lineage II isolates and divergent from the FHA AA identity from all other isolates (Figure 2). A phylogenetic tree inferred that the FHA AA sequences resulted in an overall similar tree topology to the lineage group distribution represented in the hierarchically clustered heatmap (Supplementary Figure 1A).

**Figure 3:**
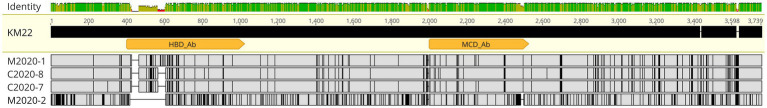
FHA alignment. MAFFT alignment of FHA protein sequences from Bb isolates M2020-1, C2020-8, C2020-7, and M2020-2. FHA from strain KM22 served as the reference sequence. Orange arrows indicate the FHA region corresponding to residues 400–1,025 used to generate polyclonal α-FHA antibodies that recognize the HBD and the region corresponding to residues 2,000–2,525 used to generate polyclonal α-FHA antibodies that recognize the MCD. In the aligned sequences, vertical black lines represent residues that differ from the KM22 FHA reference. The top bar represents mean pairwise identity over all pairs in that column of the alignment; green is 100% identical, yellow is 30 - <100% identical, and red is <30% identical. The height of the bar represents conservation of sequence at that position; a lower height indicates low sequence conservation at that position.

Given that primate isolates with the same ST harbored identical *fhaB* gene sequences, the FHA protein sequence from isolates M2020-1 (ST111), C2020-7 (ST33), C2020-8 (ST109), and M2020-2 (ST67) were used for further analyses. Protein alignments demonstrated that the AA differences compared to KM22 FHA for isolate M2020-2 (ST67), as well as all lineage II isolates, is observed throughout the whole protein sequence and not isolated to specific domains (Supplementary Figure 1B; Figure 3). While the FHA protein sequence from M2020-1 (ST111), C2020-7 (ST33), and C2020-8 (ST109) is more similar to KM22 than the FHA protein sequence from M2020-2 (ST67), there are AA differences compared to KM22 throughout the protein sequence with most located toward the C-terminal prodomain, which is removed during processing (Jungnitz et al., 1998; Coutte et al., 2001; Mazar and Cotter, 2006) (Figure 3).

Further focusing on specific domains, many AA differences compared to KM22 were observed for isolates M2020-1 (ST111), C2020-7 (ST33), C2020-8 (ST109), and M2020-2 within the Heparin Binding Domain (HBD) corresponding to residues 400–1,025 (Figure 3). Similarly, several AA differences compared to KM22 were observed for isolates M2020-1 (ST111), C2020-7 (ST33), C2020-8 (ST109), and M2020-2 (ST67) within the Mature C-terminal Domain (MCD) corresponding to residues 2,000–2,525. More AA differences compared to KM22 within both domains were observed for M2020-2 (ST67) (Figure 3). These observed AA differences led us to test whether antibodies raised against the HBD or the MCD of FHA from KM22 would recognize FHA from these isolates.

**Figure 4:**
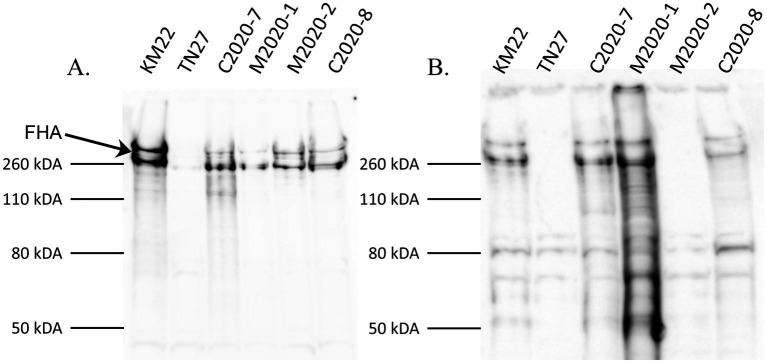
Western blot analysis using α-MCD and α-HBD KM22 antibodies. Western blot analysis of whole-cell bacterial lysates from isolates KM22, TN27 (*ΔfhaB*), C2020-7, M2020-1, M2020-2, and C2020-8. Membranes were probed with α-MCD **(A)** and α-HBD **(B)** antibodies. Sizes are indicated in kilodaltons on the left and the large ~250 kDa polypeptides indicative of multiple processed FHA proteins are indicated.

Western blot analysis of whole-cell lysates of KM22, TN27 (KM22 containing an in-frame deletion of the *fhaB* gene) (Nicholson et al., 2009), C2020-7, M2020-1, M2020-2, and C2020-8 revealed the presence of several large ~250 kDa polypeptides indicative of multiple processed FHA proteins (Delisse-Gathoye et al., 1990; Domenighini et al., 1990; Coutte et al., 2001; Mazar and Cotter, 2006; Jurnecka et al., 2018) in all isolates except TN27, when using α-FHA antibodies generated against the MCD of KM22 (Figure 4A). In contrast, western blot analysis using α-FHA antibodies generated against the HBD of KM22 revealed the presence of several processed FHA proteins of ~250 kDa only in whole-cell lysates of KM22, C2020-7, M2020-1, and C2020-8 and no FHA proteins of ~250 kDa in whole-cell lysates of TN27 and M2020-2 (Figure 4B). These data demonstrate that antibodies raised against the MCD of FHA from KM22 recognize FHA from these isolates despite AA differences compared to KM22, while antibodies raised against the HBD of FHA from KM22 do not recognize FHA from M2020-2.

The nucleotide sequence identity for *prn*, which encodes for PRN, ranged from 66.82 to 99.56%, and similar to *fhaB* sequences, primate isolates with the same ST harbored identical *prn* gene sequences (Table 2). The *prn* nucleotide sequences from lineage II baboon isolates M2020-2 and M2020-4 (both ST67) were identical and had the lowest observed identity compared to KM22 at 66.82%. The low *prn* gene sequence identity observed for the baboon lineage II isolates M2020-2 and M2020-4 suggests that the gene identified as *prn* in these isolates might not be a pertactin orthologue. Given that the gene identified as *prn* in these isolates was the only potential *prn* gene identified by BLASTN search methods, it was included in this and subsequent analyses. The *prn* nucleotide sequences from the 13 ST109 rhesus monkey isolates were identical to each other. The *prn* nucleotide sequence from rhesus monkey isolate C2020-7 (ST33) was slightly different compared to the other ST109 rhesus monkey isolates (Table 2).

**Figure 5:**
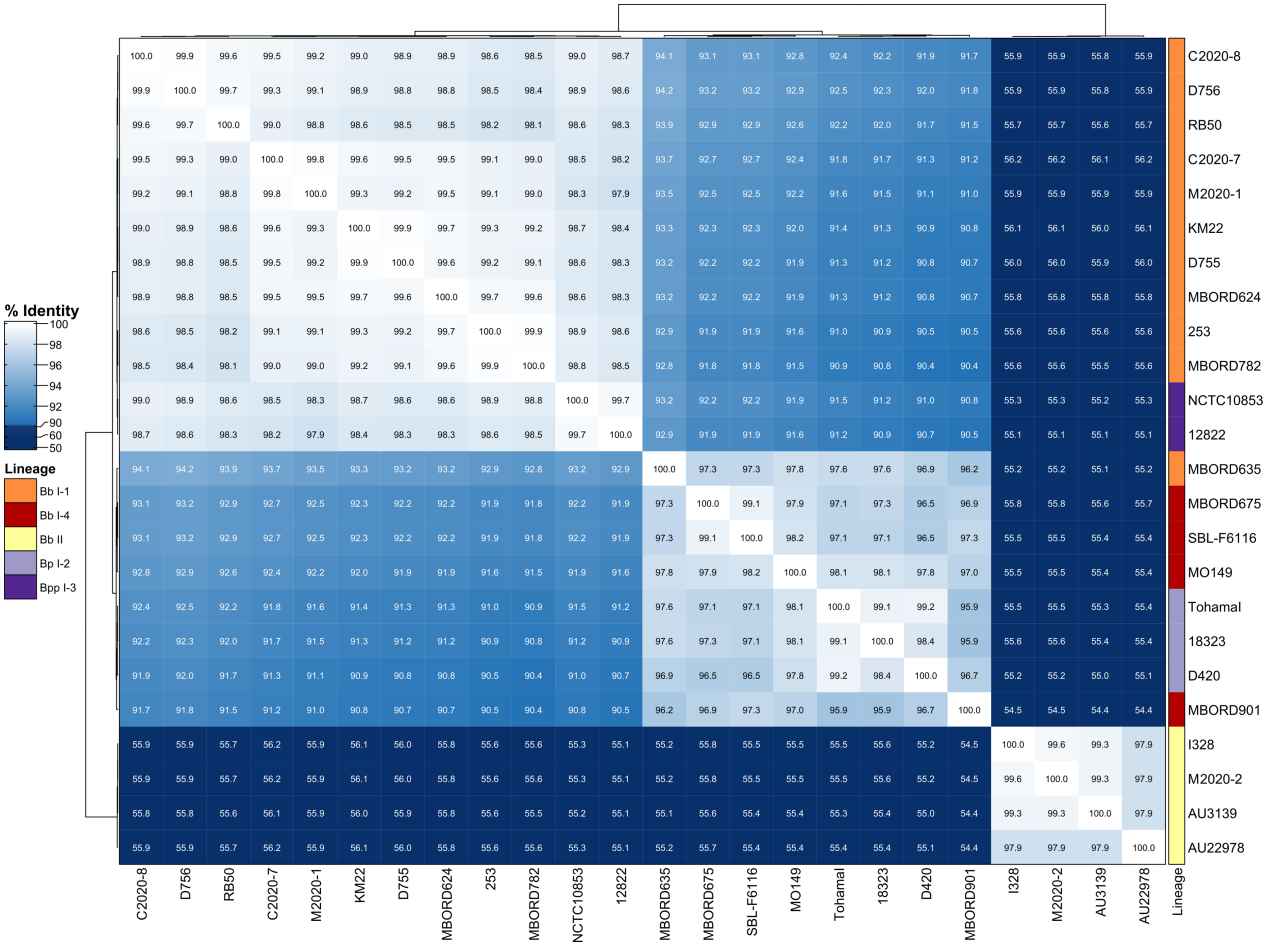
Hierarchically clustered heatmap displaying the relatedness of *Bordetella* isolates based on PRN AA identity. Pairwise AA percent identity of unique PRN sequences from isolate genomes containing a full-length *prn* gene was determined and used to generate a distance matrix heatmap clustered by hierarchical clustering using a complete linkage method with Euclidean distance. Isolate names are provided at the bottom and right side of heatmap. PRN AA identity is represented using the color scale shown left of heatmap. Dendrograms are on the left and top sides of the heat map. Lineage designations *B. bronchiseptica* (Bb I-1, Bb I-4, Bb II), *B. parapertussis* (Bpp I-3), and *B. pertussis* (Bp I-2) are shown alongside the isolate names on the right side of the heatmap colored according to the legend shown left of heatmap.

Similar to the correlation between FHA AA identity and bordetellae lineage, hierarchical clustering of primate and classical bordetellae isolates based on pairwise PRN AA identity, indicated a correlation between PRN AA identity and bordetellae lineage. The PRN AA identity is conserved among the Bb lineage II isolates and divergent from the PRN AA identity from all other isolates (Figure 5). A phylogenetic tree inferred from the PRN AA sequences resulted in an overall tree topology that correlated with the lineage group distribution represented in the hierarchically clustered heatmap (Supplementary Figure 2A).

**Figure 6:**
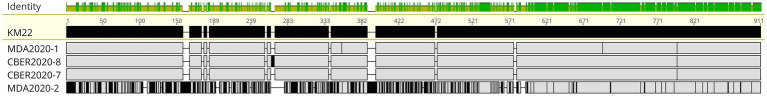
PRN alignment. MAFFT alignment of PRN protein sequences from Bb isolates M2020-1, C2020-8, C2020-7, and M2020-2. FHA from KM22 served as the reference sequence. In the aligned sequences, vertical black lines represent residues that differ from the KM22 FHA reference. The top bar represents mean pairwise identity over all pairs in that column of the alignment; green is 100% identical, yellow is 30 - <100% identical, and red is <30% identical. The height of the bar represents conservation of sequence at that position; a lower height indicates low sequence conservation at that position.

PRN sequences from isolates M2020-1 (ST111), C2020-7 (ST33), C2020-8 (ST109), and M2020-2 (ST67) were used in alignments and demonstrated that while AA differences compared to KM22 PRN were not located within specific domains, greater sequence diversity was observed within the first two-thirds of the protein and more sequence conservation was observed toward the C-terminal for isolate M2020-2 (ST67), as well as all lineage II isolates (Supplementary Figure 2B; Figure 6).

Further evaluation of the PRN AA alignment between KM22 and M2020-2 showed that PRN from M2020-2 contains two RGD domains similar to KM22 (data not shown). However, the first RGD domain PRN from M2020-2 is located closer to the N-terminal and does not align with the first RGD domain in PRN from KM22 (data not shown). The second RGD domain PRN from M2020-2 does align with the second RGD domain in PRN from KM22 (data not shown). The second RGD domain for both proteins is located in the more conserved C-terminal end, which is not present in the mature form of PRN (Gotto et al., 1993; Charles et al., 1994). In contrast, PRN protein sequences from M2020-1 (ST111), C2020-7 (ST33), and C2020-8 (ST109) were highly similar to KM22 (Figure 6). The observed AA differences led us to test whether antibodies raised against PRN would recognize PRN from M2020-2 (ST67).

**Figure 7:**
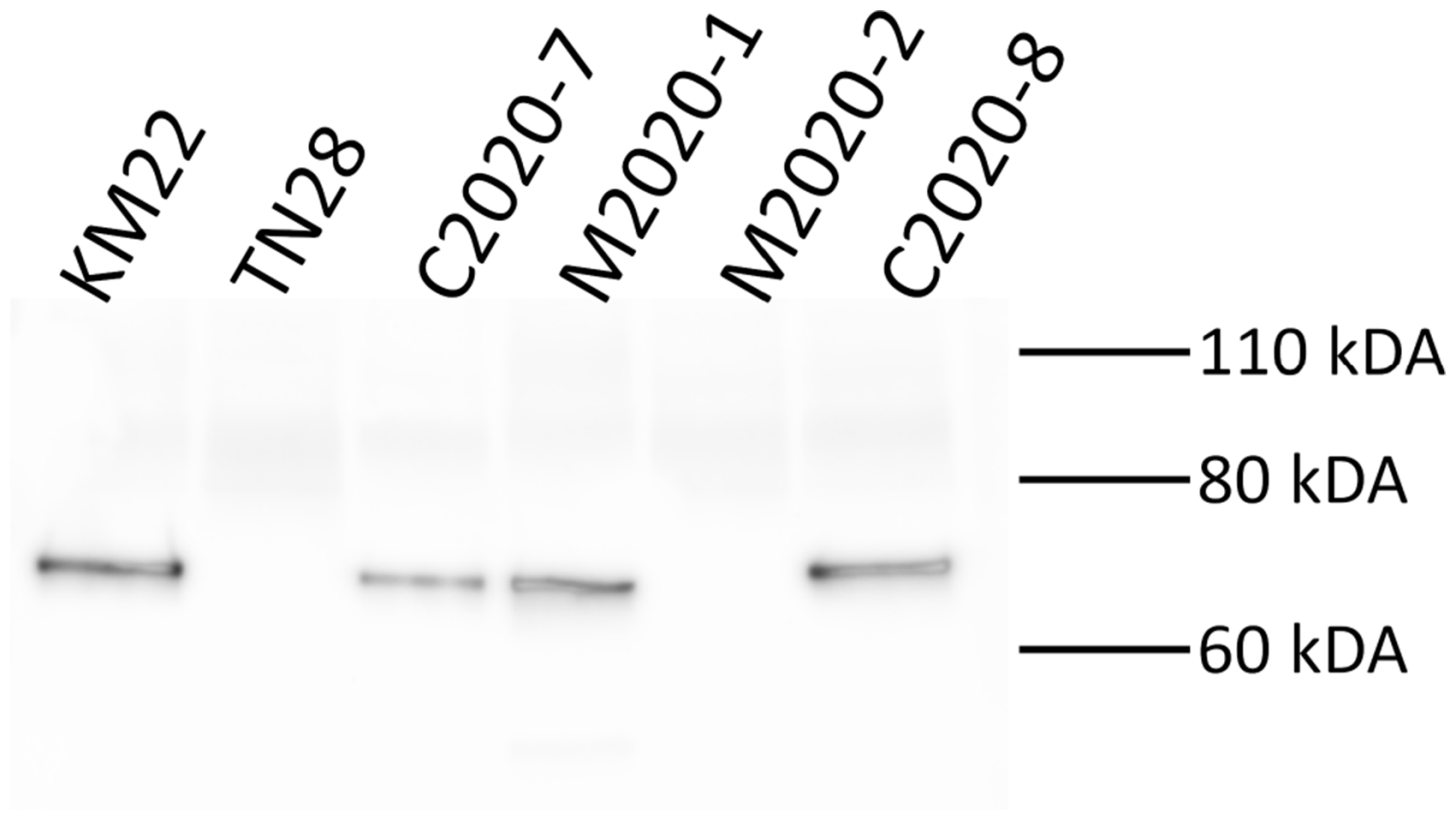
Western blot analysis using α-PRN antibodies. Western blot analysis of whole-cell bacterial lysates from isolates KM22, TN28 (*Δprn*), C2020-7, M2020-1, M2020-2, and C2020-8. Membranes were probed with α-PRN antibodies. Sizes are indicated in kilodaltons on the right.

Western blot analysis of whole-cell lysates of KM22, TN28 (KM22 containing an in-frame deletion of the *prn* gene) (Nicholson et al., 2009), C2020-7, M2020-1, M2020-2, and C2020-8 revealed the presence of a ~69 kDa polypeptide indicative of PRN only in whole-cell lysates of KM22, C2020-7, M2020-1, and C2020-8 when using α-PRN antibodies generated against the purified PRN from a Bb swine isolate (Figure 7). Contrastingly, no PRN was observed in whole-cell lysates of TN28 and M2020-2 when using α-PRN antibodies (Figure 7). These data demonstrate that antibodies raised against PRN from a Bb swine isolate recognize PRN from KM22, C2020-7, M2020-1, and C2020-8 and do not recognize PRN from M2020-2.

**Figure 8:**
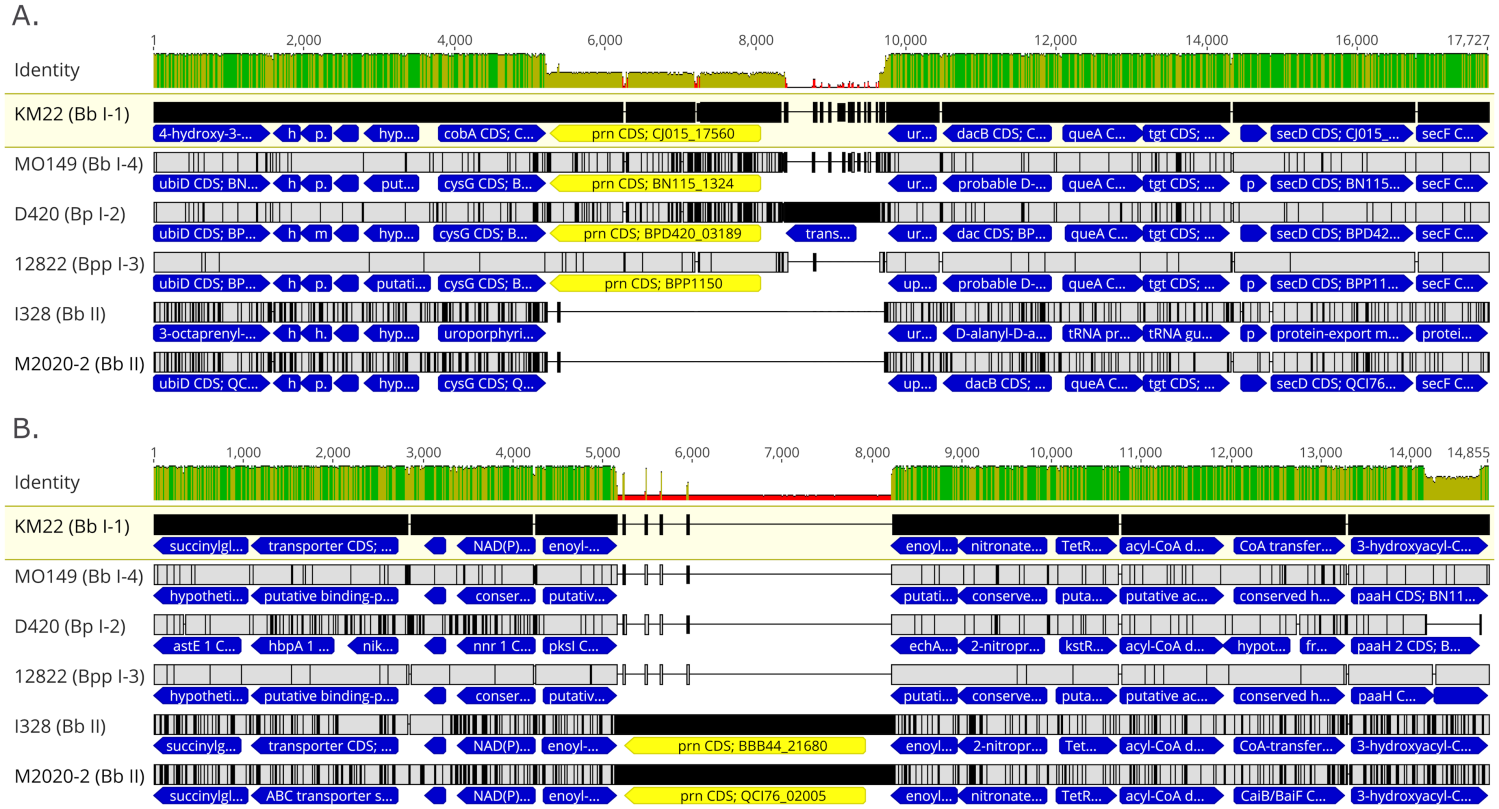
Genomic organization and alignment of the region containing the *prn* gene. MAFFT alignment of the region containing the *prn* gene for isolates KM22, MO149, D420, and 12,822 **(A)** and I328 and M2020-2 **(B)**. Bb isolate and (lineage) shown at left. In the aligned sequences, the *prn* gene (yellow) and flanking genes (blue) are shown with vertical black lines that represent residues that differ from the KM22 reference. Base pair numbers are displayed at top. The top bar represents mean pairwise identity over all pairs in that column of the alignment; green is 100% identical, yellow is 30 - <100% identical, and red is <30% identical. The height of the bar represents conservation of sequence at that position; a lower height indicates low sequence conservation at that position.

PRN repeat region typing is a discriminatory method used for genotyping Bb isolates based on the frequency of GGXXP and PQP repeat regions (Register, 2004; Register, 2001; Boursaux-Eude and Guiso, 2000). The regions containing GGXXP and PQP repeats could not be amplified *in silico* using PCR typing schemes described by Register (2001) and Register (2004) for lineage II isolates M2020-2 and M2020-4 due to lack of sequence conservation indicating that attempts to obtain PCR amplicons of these regions would likely be unsuccessful. Further *in silico* analysis of these regions found no GGXXP in the PRN sequence in any lineage II isolates (M2020-2, M2020-4, I328, I124, AU3139, or AU22978) and PQP was found only once in the PRN of these isolates. In addition to having a divergent *prn* gene sequence, the *prn* gene is located in a different genomic region in lineage II isolates compared to lineage I Bb, Bp, and Bpp isolates (Figure 8). As depicted in Figure 8, the *prn* gene is located in between predicted siroheme synthase (*cobA* /*cysG*) and uracil phosphoribosyltransferase (*upp*) genes for lineage I Bb isolates KM22 (lineage I-1), MO149 (lineage I-4), Bp D420 (lineage I-2), and Bpp 12,822 (lineage I-3) (Figure 8A). Bp D420 (lineage I-2) harbors a predicted transposase between *prn* and the *upp* gene (Figure 8A). There is no predicted gene located between these genes or within the similar genomic region in lineage II isolates I328 and M2020-2 (Figure 8B), with the flanking genes located directly adjacent to each other. In contrast, the *prn* gene is located in between two genes predicted to encode for enoyl-CoA hydratase for lineage II isolates I328 and I328 and M2020-2 (Figure 8B). There is no predicted gene located between the two genes predicted to encode for enoyl-CoA hydratase or within the similar genomic region in lineage I isolates (Figure 8A); the enoyl-CoA hydratase genes are directly adjacent to one another in lineage I isolates.

### Comparative analysis of type III and type VI secretion systems

Focusing on type III secretion system (T3SS) genes, nucleotide sequence identity for *bopB* ranged from 90.27 to 100%, *bopD* ranged from 90.87 to 100%, *bopN* ranged from 93.08 to 99.91%, *bsp22* ranged from 93.85 to 100%, *bteA* ranged from 95.27 to 99.95% (Table 2). Primate isolates with the same ST harbored identical *bopB*, *bopD*, *bopN*, and *bteA* gene sequences. However, the *bsp22* nucleotide sequences from all rhesus monkey isolates were identical to the reference KM22 sequence and baboon isolates with the same ST harbored identical *bsp22* gene sequences (Table 2).

The entire type VI secretion system (T6SS) locus was absent in the genomes of lineage II baboon isolates M2020-2, M2020-4; however, genes within the T6SS locus were highly conserved in all other lineage I primate isolates. The T6SS genes *vasA* and *clpV* listed in Table 2 reflect the observed conservation among all T6SS genes (Supplementary Table 2). The nucleotide sequence identity compared to KM22 for *vasA* ranged from 99.63 to 100% % and the nucleotide sequence identity for *clpV* ranged from 99.89 to 99.96% relative to KM22 among the genomes in which a complete *clpV* gene sequence could be compared (Table 2). The *clpV* gene was truncated at the end of contig for several genomes thus limiting comparative analysis. Lineage I primate isolates with the same ST harbored identical *vasA* gene sequences (Table 2). Additionally, lineage I primate isolates with the same ST harbored identical gene sequences for all T6SS genes for which gene sequences could be compared (Supplementary Table 2). T6SS-4 and T6SS-6 were the most conserved T6SS genes among all isolates and were 100% identical to the KM22 reference orthologue (Supplementary Table 2).

### Diversity among the fimbriae and adhesin genes

Next, we investigated the nucleotide diversity of predicted fimbriae and adhesin genes among the primate isolates. The nucleotide sequence identity for each isolate relative to the KM22 orthologue is shown in Table 3. Isolates M2020-2 and M2020-4 did not harbor genes *fimN*, *fimbrial protein 1*, *bcfA/putative adhesin 1*, *putative adhesin 2* (Table 3 and Supplementary Table 2). All other predicted fimbriae and adhesin genes harbored by isolates M2020-2 and M2020-4 had the lowest sequence identity observed (Table 3; Supplementary Table 2). Genes *fim2-like* and *putative-adhesin 2* were absent from RB50. Overall, a high degree of conservation was observed for predicted fimbriae and adhesin genes among lineage I baboon isolates M2020-1, M2020-5, and M2020-3 and rhesus monkey isolates C2020-1, C2020-2, C2020-3, C2020-4, C2020-5, C2020-8, C2020-9, C2020-10, C2020-11, C2020-12, C2020-13, C2020-14, and C2020-15. The nucleotide sequence for genes *fim2*, *fim3*, *fimC*, *fimD*, *fimbrial protein 2*, and *putative-adhesin 2* for rhesus monkey isolate C2020-7 were slightly different compared to the other lineage I baboon and rhesus monkey isolates (Table 3; Supplementary Table 2).

**Table 3:** Fimbriae and adhesin genes.

Isolate (lineage)	ST^a^	*fim2* ^b^	*fim2-like*	*fim3*	*fimA*	*fimB*	*fimC*	*fimD*	*fimN*	*fimX*	*fp1* ^d^	*fp2* ^e^	*bcfA*^f^ *put-ad1*	*put-ad2* ^g^
M2020-4 (II)	67	87.52	82.51	93.62	94.22	95.39	93.94	94.61	NP	87.95	NP	95.71	NP	NP
M2020-2 (II)	67	87.52	82.51	93.62	94.22	95.39	93.94	94.61	NP	87.95	NP	95.71	NP	NP
M2020-1 (I)	111	99.84	100	99.68	99.83	99.52	99.35	99.45	99.68	100	93.33	99.83	99.86	99.88
M2020-5 (I)	111	99.84	100	99.68	99.83	99.52	99.35	99.45	99.68	100	93.33	99.83	99.86	99.88
M2020-3 (I)	111	99.84	100	99.68	99.83	99.52	99.35	99.45	99.68	100	93.33	99.83	99.86	99.88
C2020-8 (I)	109	99.84	100	99.68	99.83	99.64	99.43	99.73	100	100	93.33	99.67	99.79	99.95
C2020-1 (I)	109	99.84	100	99.68	99.83	99.64	99.43	99.73	100	100	93.33	99.67	99.79	99.95
C2020-2 (I)	109	99.84	100	99.68	99.83	99.64	99.43	99.73	100	100	93.33	99.67	99.79	99.95
C2020-3 (I)	109	99.84	100	99.68	99.83	99.64	99.43	99.73	100	100	93.33	99.67	99.79	99.95
C2020-4 (I)	109	99.84	100	99.68	99.83	99.64	99.43	99.73	100	100	93.33	99.67	99.79	99.95
C2020-5 (I)	109	99.84	100	99.68	99.83	99.64	99.43	99.73	100	100	93.33	99.67	99.79	99.95
C2020-9 (I)	109	99.84	100	99.68	99.83	99.64	99.43	99.73	100	100	93.33	99.67	99.79	99.95
C2020-10 (I)	109	99.84	100	99.68	99.83	99.64	99.43	99.73	100	100	93.33	99.67	99.79	99.95
C2020-11 (I)	109	99.84	100	99.68	99.83	99.64	99.43	99.73	100	100	93.33	99.67	99.79	99.95
C2020-12 (I)	109	99.84	100	99.68	99.83	99.64	99.43	99.73	100	100	93.33	99.67	99.79	99.95
C2020-13 (I)	109	99.84	100	99.68	99.83	99.64	99.43	99.73	100	100	93.33	99.67	99.79	99.95
C2020-14 (I)	109	99.84	100	99.68	99.83	99.64	99.43	99.73	100	100	93.33	99.67	99.79	99.95
C2020-15 (I)	109	99.84	100	99.68	99.83	99.64	99.43	99.73	100	100	93.33	99.67	99.79	99.95
C2020-7 (I)	33	99.69	100	99.84	99.83	99.64	99.58	99.64	100	100	93.33	99.83	99.79	99.93
RB50 (I)	12	99.84	NP^c^	99.84	99.83	99.64	99.39	99.73	88.82	99.83	NP	99.67	99.83	NP
KM22 (I)	7	100	100	100	100	100	100	100	100	100	100	100	100	100

a Sequence type (ST) designation determined by BIGSdb-Pasteur database hosted by the Institut Pasteur (https://bigsdb.pasteur.fr).

b Percent identity for each gene was determined for each isolate relative to the KM22 orthologue.

c Gene not present (NP) in genome sequence.

d Fimbrial protein 1(fp1).

e Fimbrial protein 2 (fp2).

f BcfA/putative-adhesin 1 (put-ad1).

g Putative-adhesin 2 (put-ad2).

**Figure 9:**
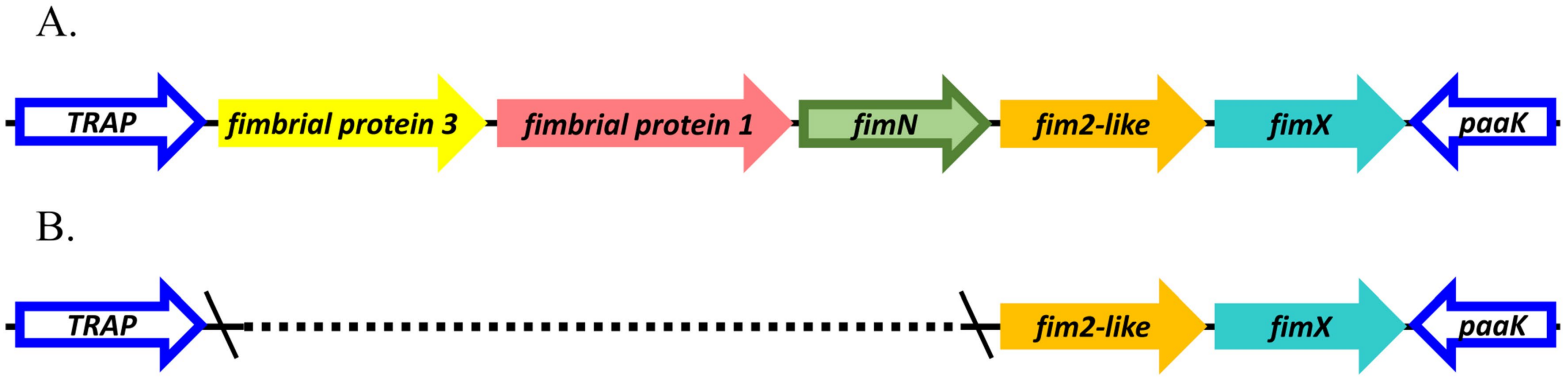
Organization of *fimNX* locus. Predicted fimbrial protein genes are represented as arrows; gene names refer to names used in Table 3 and are color-coded by nucleotide sequence identity. *fim*NX locus for isolates **(A)** M2020-1, M2020-5, M2020-3, C2020-8, C2020-1, C2020-2, C2020-3, C2020-4, C2020-5, C2020-9, C2020-10, C2020-11, C2020-12, C2020-13, C2020-14, C2020-15, C2020-7 and **(B)** M2020-4 and M2020-2.

Focusing on the *fimNX* locus, all lineage I baboon isolates M2020-1, M2020-5, and M2020-3 and all lineage I rhesus monkey isolates C2020-1, C2020-2, C2020-3, C2020-4, C2020-5, C2020-8, C2020-9, C2020-10, C2020-11, C2020-12, C2020-13, C2020-14, C2020-15, and C2020-7 harbored five predicted fimbrial genes (Figure 9). These predicted fimbrial genes include *fimbrial protein 3*, *fimbrial protein 1*, *fimN*, *fim2-like*, and *fimX* (Figure 9A). In contrast, lineage II baboon isolates M2020-4 and M2020-2 harbored only two predicted fimbrial genes *fim2-like* and *fimX* (Figure 9B). Despite the diversity in the number of genes harbored, the *fimNX* locus was located in the same genomic location flanked by tripartite ATP-independent periplasmic (TRAP) transporter and phenylacetate-CoA ligase gene (*paaK*) genes in all primate isolates, as well as in RB50 and KM22 (Figures 9A,B and data not shown).

### Comparative analysis of the *cya*, *ptp*, and loci

Genes *cyaA*, *cyaB*, *cyaC*, *cyaD*, and *cyaE* of the *cya* operon were absent from the genomes of lineage II baboon isolates M2020-2 and M2020-4, however, these genes were highly conserved among all other lineage I primate isolates. Additionally, the *cyaX* gene was present and highly conserved in all primate isolates (Table 4). It has been demonstrated that the *cya* operon, which contains genes that encode, activate, and secrete Adenylate cyclase toxin, was replaced by an operon predicted to encode peptide transport (*ptp*) proteins in Bb ST37 isolates (Buboltz et al., 2008). The *ptp* operon was flanked by *gabD* and *cyaX* genes in Bb ST37 isolates, and the genes were reported as highly conserved among ST37 isolates (Buboltz et al., 2008). Similarly, genes within the *cya* operon were replaced by the *ptp* operon, flanked by *gabD* and *cyaX* genes, in lineage II isolates M2020-4, M2020-2, 99-R-0433, and AU3139 (Table 5). Neither the *cya* operon or the *ptp* operon was harbored by lineage II isolates I124 and I328. Lineage II isolate AU3139 harbored both the *cya* operon or the *ptp* operon, however, the *cyaA* gene was predicted to be non-functional due to a frameshift (Table 5).

**Table 4:** *cyaA* operon and the O-antigen serotype.

Isolate (Lineage)	ST^a^	*cyaA*	*cyaB*	*cyaC*	*cyaD*	*cyaE*	*cyaX*	O-Antigen (*in silico* PCR)^c^
M2020-4 (II)	67	NP^b^	NP	NP	NP	NP	95.6	NF
M2020-2 (II)	67	NP	NP	NP	NP	NP	95.6	NF
M2020-1 (I)	111	99.44	99.95	98.81	100	99.72	100	O2
M2020-5 (I)	111	99.44	99.95	98.81	100	99.72	100	O2
M2020-3 (I)	111	99.44	99.95	98.81	100	99.72	100	O2
C2020-8 (I)	109	99.77	99.91	98.98	99.92	99.65	100	O2
C2020-1 (I)	109	99.77	99.91	98.98	99.92	99.65	100	O2
C2020-2 (I)	109	99.77	99.91	98.98	99.92	99.65	100	O2
C2020-3 (I)	109	99.77	99.91	98.98	99.92	99.65	100	O2
C2020-4 (I)	109	99.77	99.91	98.98	99.92	99.65	100	O2
C2020-5 (I)	109	99.77	99.91	98.98	99.92	99.65	100	O2
C2020-9 (I)	109	99.77	99.91	98.98	99.92	99.65	100	O2
C2020-10 (I)	109	99.77	99.91	98.98	99.92	99.65	100	O2
C2020-11 (I)	109	99.77	99.91	98.98	99.92	99.65	100	O2
C2020-12 (I)	109	99.77	99.91	98.98	99.92	99.65	100	O2
C2020-13 (I)	109	99.77	99.91	98.98	99.92	99.65	100	O2
C2020-14 (I)	109	99.77	99.91	98.98	99.92	99.65	100	O2
C2020-15 (I)	109	99.77	99.91	98.98	99.92	99.65	100	O2
C2020-7 (I)	33	99.41	99.81	98.98	99.92	99.58	99.89	O2
RB50 (I)	12	99.43	99.81	98.81	99.92	99.58	99.89	O1
KM22 (I)	7	100	100	100	100	100	100	O2

a Sequence type (ST) designation determined by BIGSdb-Pasteur database hosted by the Institut Pasteur (https://bigsdb.pasteur.fr).

b Gene not present (NP) in genome sequence.

c Genes encoding an O-antigen serotype O1, O2 or not found (NF) in genome sequence based on *in silico* PCR using primers from Buboltz et al. (2009a).

**Table 5:** *cyaA* or *ptp* operon.

Lineage II Strains	*cya* or *ptp* operon
M2020-4	*ptp*
M2020-2	*ptp*
99-R-0433	*ptp*
AU22978	Both; but possibly non-functional due to frameshift in *cyaA*
AU3139	*ptp*
I124	Neither
I328	Neither

### Diversity among the *wbm* loci encoding O-antigen serotypes

The *wbm* locus contains genes required for expression of one of three antigenically distinct O-antigen types defined as O1, O2, or O3 serotype (Vinogradov et al., 2010; Preston et al., 1999; Hester et al., 2013; Buboltz et al., 2009a). An *in silico* PCR test based on PCR typing schemes described by Buboltz et al. (2009a) was used to screen the genomes of all primate isolates, RB50, and KM22. The O-antigen type O2 was observed in all lineage I primate isolates and KM22, while the O-antigen type O1 was observed for RB50. Neither the O-antigen type O1 or O2 were found for lineage II baboon isolates M2020-4 and M2020-2 using *in silico* PCR (Table 4).

**Figure 10:**
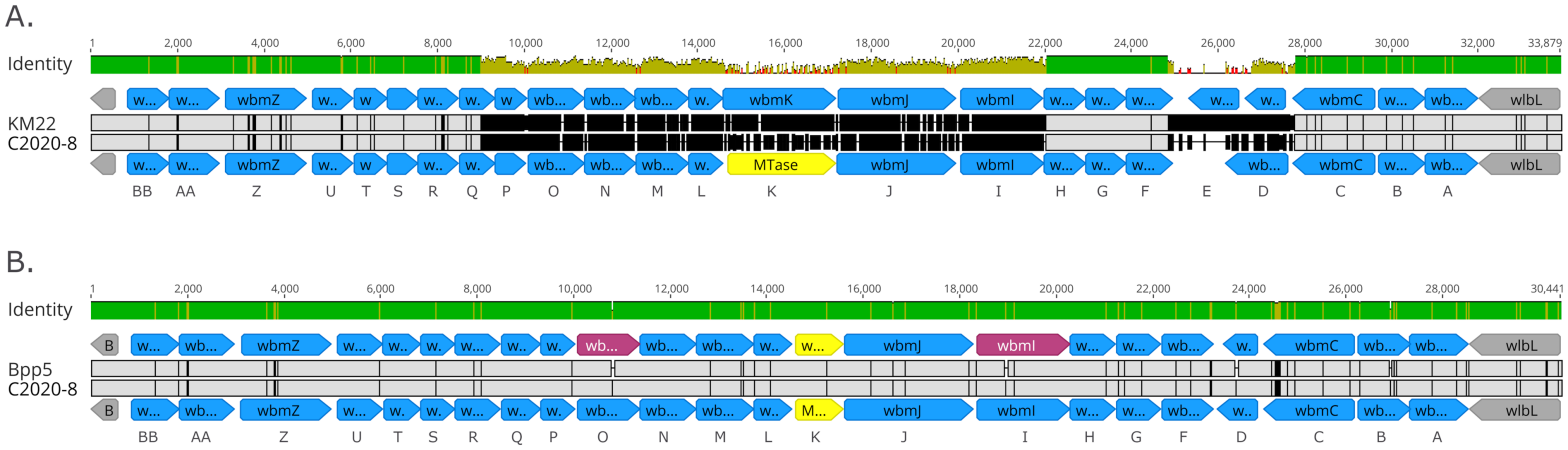
Organization of *wbm* locus from rhesus monkey Bb isolates. Alignment of the *wbm* locus of C2020-8 to KM22 serving as reference O-antigen serotype O2 **(A)** and alignment of the *wbm* locus of C2020-8 to Bpp_ov_ strain Bpp5 serving as reference **(B)**. *wbm* gene name designations are provided at bottom of each alignment. Predicted genes within *wbm* locus are represented as blue arrows and genes flanking the *wbm* locus are indicated by grey arrows. Unique gene, which is not present in O-antigen serotype O2, is indicated by yellow arrows. Pseudogenes are indicated by pink arrows. Base pair numbers are displayed above black bar at top. In the aligned sequences, vertical black lines represent residues that differ from the reference. The top bar represents mean pairwise identity over all pairs in that column of the alignment; green is 100% identical, yellow is 30 - <100% identical, and red is <30% identical. The height of the bar represents conservation of sequence at that position; a lower height indicates low sequence conservation at that position.

Further examination of the *wbm* locus for all rhesus monkey isolates revealed that while the 3′ and 5′ ends of the *wbm* locus were conserved and highly similar to a *wbm* locus encoding genes for O-antigen serotype O2, a high degree of nucleotide divergence was observed for centrally located *wbm* genes *wbmP*—*wbmI* and *wbmD* (Figure 10). As shown in Figure 10, the *wbm* locus of C2020-8, representing all rhesus monkey isolates, was more similar to the *wbm* locus of Bpp_ov_ Bpp5 than to the KM22 O-antigen serotype O2 locus (Figures 10A,B). Bpp_ov_ strain Bpp5 does not produce an O-antigen molecule due to frameshift mutations in the *wbmO* and *wbmI* genes (Hester et al., 2015). Similar to Bpp_ov_ Bpp5, *wbmE* is missing in the *wbm* locus of C2020-8 and *wbmK* is replaced by a unique gene, which encodes a methyltransferase (Figure 10B) (Hester et al., 2015). However, unlike the *wbm* locus of Bpp_ov_ Bpp5, *wbmO* and *wbmI* genes within the *wbm* locus for rhesus monkey isolates are predicted to be functional as they do not contain frameshift mutations (Figure 10B) (Hester et al., 2015).

**Figure 11:**
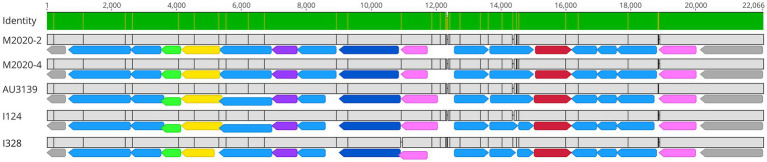
M2020-2 and M2020-4 harbor a novel O-antigen serotype. Alignment of the *wbm* locus for isolates M2020-2, M2020-4, AU3139I124, and I328. The *wbm* locus for these isolates includes 16 predicted genes, with functional annotations including predicted formyltransferase genes (green arrows), predicted aminotransferase genes (yellow arrows), predicted methyltransferase genes (purple arrows), predicted asparagine synthase genes (dark blue arrows), predicted glyceroltransferase genes (pink arrows), predicted sugar amidotransferase genes (red arrows), and other genes of undetermined function (light blue arrows). Genes flanking the *wbm* locus are indicated by grey arrows. Base pair numbers are displayed above black bar at top. In the aligned sequences, vertical black lines represent residues that differ between sequences. The top bar represents mean pairwise identity over all pairs in that column of the alignment; green is 100% identical and yellow is 30 - <100% identical.

A *wbm* locus was identified in lineage II baboon isolates M2020-2 and M2020-4; however, this *wbm* locus has very low sequence identity compared to *wbm* loci encoding for O-antigen serotypes O1, O2, or O3 (Supplementary Figure 3). The *wbm* locus identified in lineage II baboon isolates M2020-2 and M2020-4 was observed to be novel and highly similar to the *wbm* locus harbored by other lineage II isolates AU3139, I124, and I128 (Figure 11). The *wbm* locus harbored by these isolates contain 16 predicted genes that appear to encode for a novel O-antigen serotype (Figure 11). This predicted novel O-antigen locus was not harbored by all lineage II isolates evaluated. Lineage II isolate 99-R-0433 harbored a *wbm* locus with a high similarity (88%) to the *wbm* locus of strain MO149 encoding for an O-antigen serotype O3 (Vinogradov et al., 2010) and lineage II isolate AU22978 harbored a *wbm* locus with a high similarity (88%) to the *wbm* locus of strain RB50 encoding for an O-antigen serotype O1 (Buboltz et al., 2009a).

### AMR distribution

Phenotypic AMR to 10 antibiotic classes was determined, and all isolates were resistant to specific antibiotics within the β-lactam class (Table 6; Supplementary Table 3). Additionally, a high prevalence of resistance was observed for macrolide/lincosamide/streptogramin (MLSb) (95%, *n* = 18), pleuromutilin (95%, *n* = 18), and fluroquinolone (69%, *n* = 13) antibiotic classes (Table 6; Supplementary Table 3). In contrast, no isolates exhibited resistance to aminoglycoside, tetracycline, phenicol, polymyxin, sulfonamide, or glycylcycline antibiotic classes (Table 6; Supplementary Table 3). Thirteen isolates exhibited phenotypic resistance to four of the 10 antibiotic classes tested and five isolates exhibited phenotypic resistance to three of the 10 antibiotic classes tested (Supplementary Table 3).

**Table 6:** Phenotypic AMR prevalence among primate *B. bronchiseptica* isolates.

Antibiotic class	Antibiotic	Number (%)^a^
Aminoglycoside	Amikacin	0 (0%)
	Gentamicin	0 (0%)
	Neomycin	0 (0%)
	Spectinomycin	0 (0%)
β-lactam/Penicillin	Ampicillin	18/ (95%)
	Aztreonam	19/ (100%)
	Doripenem	0 (0%)
	Imipenem	0 (0%)
	Meropenem	0 (0%)
	Penicillin	18/ (95%)
	Piperacillin / tazobactam	0 (0%)
	Ticarcillin / clavulanic acid	0 (0%)
β-lactam/Cephalosporin	Cefepime	0 (0%)
	Cefotaxime	18/ (95%)
	Ceftazidime	0 (0%)
	Ceftiofur	18/ (95%)
Fluroquinolone	Ciprofloxacin	0 (0%)
	Danofloxacin	13/ (68%)
	Enrofloxacin	6 (32%)
	Levofloxacin	0 (0%)
Macrolide/Lincosamide/Streptogramin (MLSb)	Clindamycin	18/ (95%)
	Gamithromycin	0 (0%)
	Tilmicosin	0 (0%)
	Tildipirosin	0 (0%)
	Tulathromycin	0 (0%)
Phenicol	Florfenicol	0 (0%)
Pleuromutilin	Tiamulin	18/ (95%)
Polymyxin	Colistin	0 (0%)
	Polymixin B	0 (0%)
Sulfonamide	Sulfadimethoxine	0 (0%)
	Trimethoprim/Sulfamethoxazole	0 (0%)
Tetracycline	Doxycycline	0 (0%)
	Minocycline	0 (0%)
	Tetracycline	0 (0%)
Glycylcycline	Tigecycline	0 (0%)

a Number of isolates resistant to indicated antibiotic (percentage of isolates tested).

Focusing on specific antibiotics tested, isolates primarily exhibited resistance to ampicillin, aztreonam, and/or penicillin within the penicillin subclass of the β-lactam antibiotic class and cefotaxime and/or ceftiofur within the cephalosporin subclass of the β-lactam antibiotic class (Table 6; Supplementary Table 3). Resistance to the MLSb class was due to resistance to clindamycin (Table 6; Supplementary Table 3). The observed resistance to the fluroquinolone class was primarily due to resistance to danofloxacin (Table 6; Supplementary Table 3). While not exhibiting complete resistance to tilmicosin, within the MLSb class, 18 isolates exhibited intermediate resistance to tilmicosin (Supplementary Table 3). Similarly, while no isolates were observed to exhibit complete resistance to florfenicol, within the phenicol class, 17 isolates exhibited intermediate resistance to florfenicol (Supplementary Table 3).

Genomes were screened for chromosomal mutations and genes conferring AMR and only the *Bordetella*-specific *blaBOR* β-lactamase gene (Lartigue et al., 2005) was identified (Supplementary Table 3). All isolates harbored the *blaBOR* β-lactamase gene (Supplementary Table 3). The *blaBOR* β-lactamase gene from 13 rhesus monkey isolates were 100% identical to each other and 99.78% identical to the reference RB50 sequence *blaBOR* β-lactamase gene (Supplementary Table 3). The rhesus monkey isolate C2020-7 differed from the other rhesus monkey isolates and was 99.67% identical to the reference RB50 sequence (Supplementary Table 3). The lineage I baboon isolates (M2020-1, M2020-3, and M2020-5) were 100% identical to each other and 99.89% identical to the reference RB50 sequence (Supplementary Table 3). The *blaBOR* β-lactamase gene from the lineage II baboon isolates (M2020-2, M2020-4) were 100% identical to each other and 93.36% identical to the reference RB50 sequence (Supplementary Table 3). No other AMR elements were identified.

## Discussion

There is very little whole-genome data available for Bb isolates obtained from non-human primate hosts. To our knowledge, Bb strain 1,289 obtained from a monkey host, is the only publicly available genome sequence available for a Bb strain obtained from a non-human primate species (Buboltz et al., 2009b). In this report we use whole genome sequencing to evaluate the genetic diversity of Bb isolates obtained from non-human primates, five obtained from baboons and 14 from rhesus monkeys. Most of the isolates evaluated in this report represented one of the two new sequence types (STs) within Bb lineage I-1 clonal complex 6 that were identified by this study. Two baboon isolates, M2020-2 and M2020-4, were ST67 and determined to be Bb lineage II isolates. Given that the genome sequences for M2020-2 and M2020-4 were almost identical and both isolates were obtained from baboons housed together, they are likely the same isolate transmitted between the baboons. While M2020-2 and M2020-4 were isolated from the only animals known to be cohoused together prior to inclusion in this study, the possibility that other isolates were obtained from animals housed together in large groups cannot be excluded.

It has been previously demonstrated that genes encoding virulence factors and vaccine antigens are highly variable and unique to Bb lineage II isolates (Bridel et al., 2022). The data for lineage II baboon isolates M2020-2 and M2020-4 agree with these previous observations. Specially, these isolates harbored the lowest sequence identity observed across all regulatory and virulence-associated genes evaluated. Additionally, these isolates do not have the *dnt* gene, T6SS locus genes, the *cya* operon, and fimbrial genes *fimN*, *fimbrial protein 1*, *bcfA/putative adhesin 1*, and *putative adhesin 2*.

Both FHA and PRN are Bp vaccine antigens and extensive genomic diversity has been documented within the genes encoding these antigens as well as throughout the whole genome among Bp isolates (Komatsu et al., 2010; Bart et al., 2014; Weigand et al., 2017a; Weigand et al., 2017b). Hierarchical clustering of ANI values among the primate isolates evaluated in this study and additional classical bordetellae isolates highly correlated with the phylogenetic group distribution of the cgMLST-based tree reported by Bridel et al. (2022). The similar correlation between AA identity and classical bordetellae lineage for both FHA and PRN indicate that the genes encoding these proteins are core genes in which allele variation reflects phylogenetic group distribution, which is in agreement to other reports (Bridel et al., 2022; Diavatopoulos et al., 2005; Linz et al., 2016; Park et al., 2012).

While AA differences of FHA between KM22 and M2020-2, as well as all lineage II isolates, were observed throughout the whole protein sequence and not located within specific domains, antibodies raised against the MCD of FHA from KM22 recognized FHA from M2020-2, whereas antibodies raised against the HBD of FHA from KM22 did not recognize FHA from M2020-2. This is the first report demonstrating antibodies generated against a specific domain of FHA from one Bb isolate not recognizing FHA from another isolate. Due to the high AA similarity among the lineage II isolates, the data suggests that antibodies generated against FHA from a lineage I isolate could result in decreased cross-protective immunity toward FHA from a lineage II isolate.

The *prn* gene sequence identity to the KM22 *prn* was 86.16% for the baboon lineage II isolates M2020-2 and M2020-4. The low nucleotide and AA identity observed for M2020-2 and M2020-4 was observed among all the Bb lineage II isolates. Additionally, the *prn* gene was located in a different genomic region in lineage II isolates compared to lineage I Bb, Bp, and Bpp isolates. Due to the low AA identity of M2020-2 compared to KM22, and other lineage I Bb isolates, antibodies raised against PRN from a Bb swine isolate did not recognize PRN from M2020-2. While the Bb swine isolate used for the purification of PRN and antibody generation was not KM22, all swine Bb isolates studied to date are Bb lineage I and harbor extreme genomic similarity (Nicholson and Shore, 2024). Due to the high AA similarity among the lineage II isolates, the data suggests that antibodies generated against PRN from a lineage I isolate would result in no cross-protective immunity toward PRN from a lineage II isolate.

PRN belongs to the type V autotransporter protein family and contains two RGD domains (Emsley et al., 1996; Hijnen et al., 2004). Bridel et al. (2022) reported Bb lineage II isolates do not harbor a *prn* gene sequence. The *prn* gene was identified in all isolates using BLASTN searches. The gene identified and evaluated as the *prn* gene in the lineage II isolates M2020-2 and M2020-4 was the only gene hit identified in these isolates, despite the low sequence identity. However, the possibility that the gene identified as *prn* might not be *prn* but instead another gene within type V autotransporter family cannot be excluded given the low *prn* gene sequence identity and the observation that the gene was located in a different genomic region compared to lineage I Bb, Bpp, and Bp isolates.

Buboltz et al. (2008) demonstrated that the *cya* operon was replaced by the *ptp* operon in Bb lineage I ST37 isolates. We observed the *cya* operon was replaced by the *ptp* operon in several lineage II isolates expanding the phylogenetic distribution of this operon replacement. Furthermore, we observed heterogeneity within this locus among lineage II isolates. Specifically, both the *cya* and the *ptp* operons were absent in some lineage II isolates and one lineage II isolate harbored both the *cya* operon and the *ptp* operon. The data reported here greatly expands knowledge regarding phylogenetic diversity and genetic variation within this locus among Bb isolates.

Recombination-mediated horizontal gene transfer events involving O-antigen genes contribute to O-antigen variation and previous studies have characterized different O-antigen types among the classical bordetellae subspecies (Hester et al., 2013). Additionally, the genetic variation within the *wbm* locus enables *Bordetella* strains to escape immunity to different O-antigen types, thereby allowing for the circulation of different *Bordetella* strains within the same host population (Hester et al., 2013). Building on the previously reported variation within the *wbm* locus, the O-antigen locus harbored by all rhesus monkey isolates was similar to the O-antigen locus reported for Bpp_ov_ Bpp5 (Hester et al., 2015), with the exception of *wbmO* and *wbmI* genes, which were predicted to be functional as they do not contain frameshift mutations. Additionally, the *wbm* locus harbored by lineage II baboon isolates M2020-2 and M2020-4, as well as other lineage II isolates AU3139, I124, and I128, was observed to encode a novel unreported O-antigen. Collectively, the data reported here broadens the described variation within the *wbm* locus containing genes encoding for the expression of antigenically distinct O-antigen types.

Bb isolates are well known to be generally resistant to β-lactams and macrolides (Woolfrey and Moody, 1991; Kadlec and Schwarz, 2018). In fact, macrolide resistance was previously an important clinical treatment distinction between Bb and Bp before the recent occurrence of macrolide-resistant isolates of Bp (Woolfrey and Moody, 1991; Mattoo and Cherry, 2005; Ivaska et al., 2022). The phenotypic AMR observed among the primate isolates agrees with these reports, with all of the isolates exhibiting resistance to β-lactams. The high prevalence of resistance observed for the macrolide/lincosamide/streptogramin (MLSb) class was due to resistance to clindamycin, a lincosamide antibiotic. While structurally distinct, lincosamide and macrolide antibiotics are grouped together in the same class due to a shared mechanism of action (Canu and Leclercq, 2009). A high prevalence of resistance was observed for pleuromutilin and fluroquinolone antibiotic classes, which has not been previously reported for Bb isolates. Tetracyclines are commonly used to treat Bb veterinary infections due to a high level of sensitivity reported (Kadlec and Schwarz, 2018). Consistent with these previous reports, no isolates exhibited resistance to tetracycline. The *Bordetella*-specific *blaBOR* β-lactamase gene (Lartigue et al., 2005), harbored by all primate Bb isolates, was the only AMR gene identified. Unfortunately, with the exception of the *blaBOR* β-lactamase gene, the occurrence of elevated MICs among the Bb isolates toward other antibiotic classes could not be clearly associated with specific chromosomal mutations or AMR genes, which is consistent with prior reports (Kadlec and Schwarz, 2018; Pruller et al., 2015).

In conclusion, this study presents genomic and phenotypic data for several non-human primate Bb isolates. Combined, the data in this report expands the known diversity of Bb isolates and highlights distinctive traits of Bb isolates between different lineages as well as distinctive traits among Bb isolates within the same lineage.

## Data Availability

The datasets presented in this study can be found in online repositories. The names of the repository/repositories and accession number(s) can be found in the article/Supplementary material.

## Footnotes

^1^ https://www.bioinformatics.babraham.ac.uk/projects/fastqc/

^2^ https://sourceforge.net/projects/bbmap/

^3^ https://bigsdb.pasteur.fr

^4^ https://www.geneious.com

## Supplementary material

SUPPLEMENTARY FIGURE 1 FHA phylogenetic tree and alignment. **(A)** Phylogenetic tree inferred from unique FHA AA sequences from analyzed *Bordetella* isolates containing a full-length *fhaB* gene used to build a maximum-likelihood phylogenetic tree with FastTree (v2.11) and iTOL (Letunic and Bork, 2024; Price et al., 2010). Support values are shown on each branch, and isolate names are color coded according to legend shown left of the heatmap. **(B)** MAFFT alignment of the unique FHA AA sequences from analyzed *Bordetella* isolates containing a full-length *fhaB* gene ordered by lineage. FHA from Bb strain KM22 served as the reference sequence. In the aligned sequences, vertical black lines represent residues that differ from the KM22 FHA reference. The top bar represents mean pairwise identity over all pairs in that column of the alignment; green is 100% identical, yellow is 30 - <100% identical, and red is <30% identical. The height of the bar represents conservation of sequence at that position; a lower height indicates low sequence conservation at that position.

SUPPLEMENTARY FIGURE 2 PRN alignment and phylogenetic tree. **(A)** Phylogenetic tree inferred from non-redundant PRN AA sequences from analyzed *Bordetella* isolates containing a full-length *prn* gene used to build a maximum-likelihood phylogenetic tree with FastTree (v2.11) and iTOL (Letunic and Bork, 2024; Price et al., 2010). Support values are shown on each branch, and isolate names are color coded according to legend shown left of the heatmap. **(B)** MAFFT alignment of the unique PRN AA sequences from analyzed *Bordetella* isolates containing a full-length *prn* gene ordered by lineage. PRN from Bb strain KM22 served as the reference sequence. In the aligned sequences, vertical black lines represent residues that differ from the KM22 FHA reference. The top bar represents mean pairwise identity over all pairs in that column of the alignment; green is 100% identical, yellow is 30 - <100% identical, and red is <30% identical. The height of the bar represents conservation of sequence at that position; a lower height indicates low sequence conservation at that position.

SUPPLEMENTARY FIGURE 3 M2020-2 and M2020-4 do not harbor O-antigen serotype O1, O2, or O3. Alignment of the *wbm* locus M2020-2 to RB50 serving as reference O-antigen serotype O1 **(A)**, *wbm* locus M2020-2 to KM22 serving as reference O-antigen serotype O2 **(B)**, and *wbm* locus M2020-2 to MO149 serving as reference O-antigen serotype O3 **(C)**. M2020-2 and M2020-4 sequences are 100% identical over the region therefore only M2020-2 sequence is used in alignments. Predicted genes within the *wbm* locus are represented as blue arrows and genes flanking the *wbm* locus are indicated by grey arrows. Base pair numbers are displayed above black bar at top. In the aligned sequences, vertical black lines represent residues that differ from the reference. The top bar represents mean pairwise identity over all pairs in that column of the alignment; green is 100% identical, yellow is 30 - <100% identical, and red is <30% identical. The height of the bar represents conservation of sequence at that position; a lower height indicates low sequence conservation at that position.
