# Supplementary figures and images for: Genetic diversity of Bordetella bronchiseptica isolates obtained from primates
Source: Front Microbiol. 2025 Jun 26;16:1571660. doi: 10.3389/fmicb.2025.1571660 (PMC12243275; doi:10.3389/fmicb.2025.1571660)

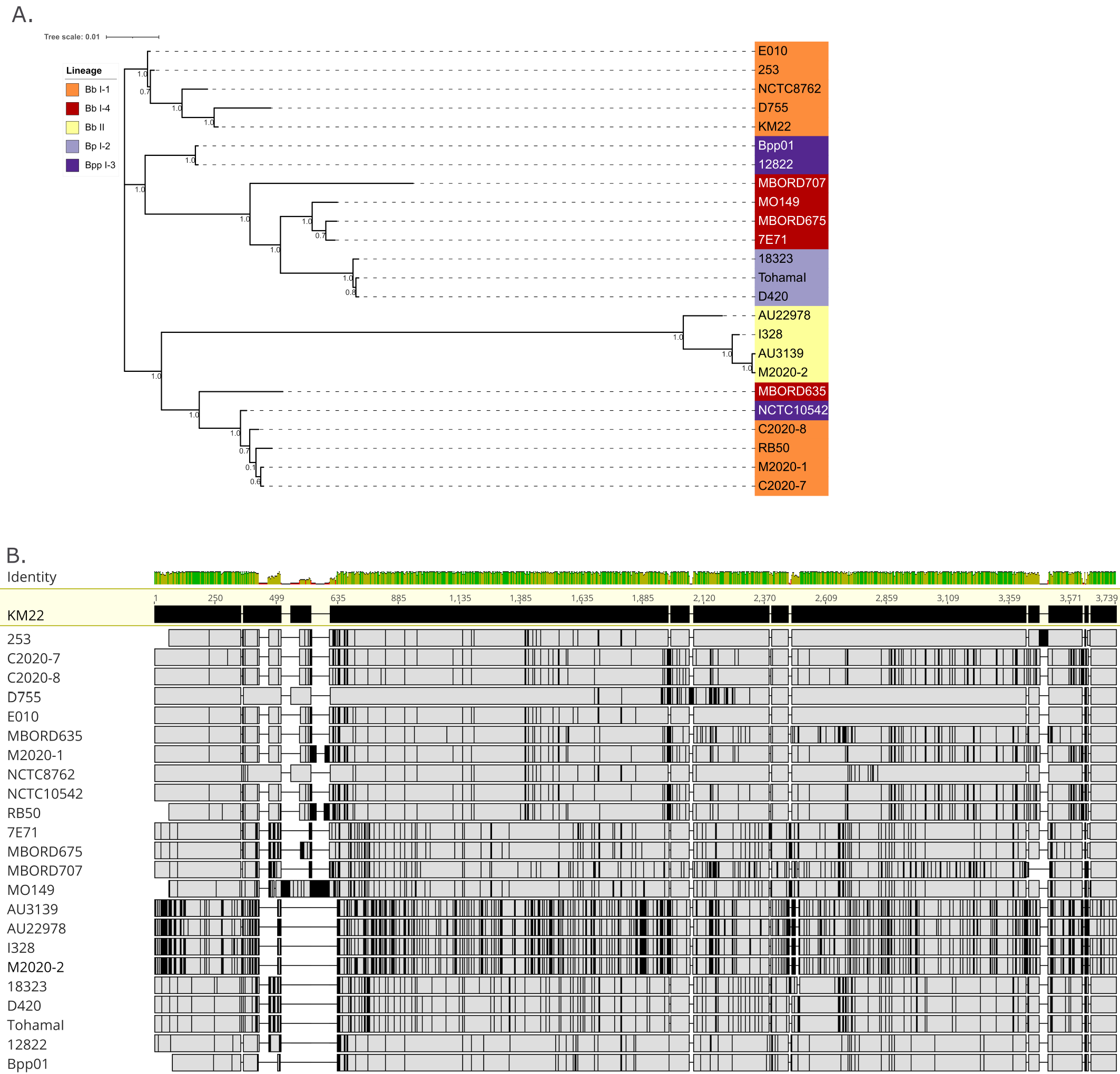

Supplement: SUPPLEMENTARY FIGURE 1 — FHA phylogenetic tree and alignment. (A) Phylogenetic tree inferred from unique FHA AA sequences from analyzed Bordetella isolates containing a full-length fhaB gene used to build a maximum-likelihood phylogenetic tree with FastTree (v2.11) and iTOL (Letunic and Bork, 2024; Price et al., 2010). Support values are shown on each branch, and isolate names are color coded according to legend shown left of the heatmap. (B) MAFFT alignment of the unique FHA AA sequences from analyzed Bordetella isolates containing a full-length fhaB gene ordered by lineage. FHA from Bb strain KM22 served as the reference sequence. In the aligned sequences, vertical black lines represent residues that differ from the KM22 FHA reference. The top bar represents mean pairwise identity over all pairs in that column of the alignment; green is 100% identical, yellow is 30 - <100% identical, and red is <30% identical. The height of the bar represents conservation of sequence at that position; a lower height indicates low sequence conservation at that position. [file Image_1.tif]

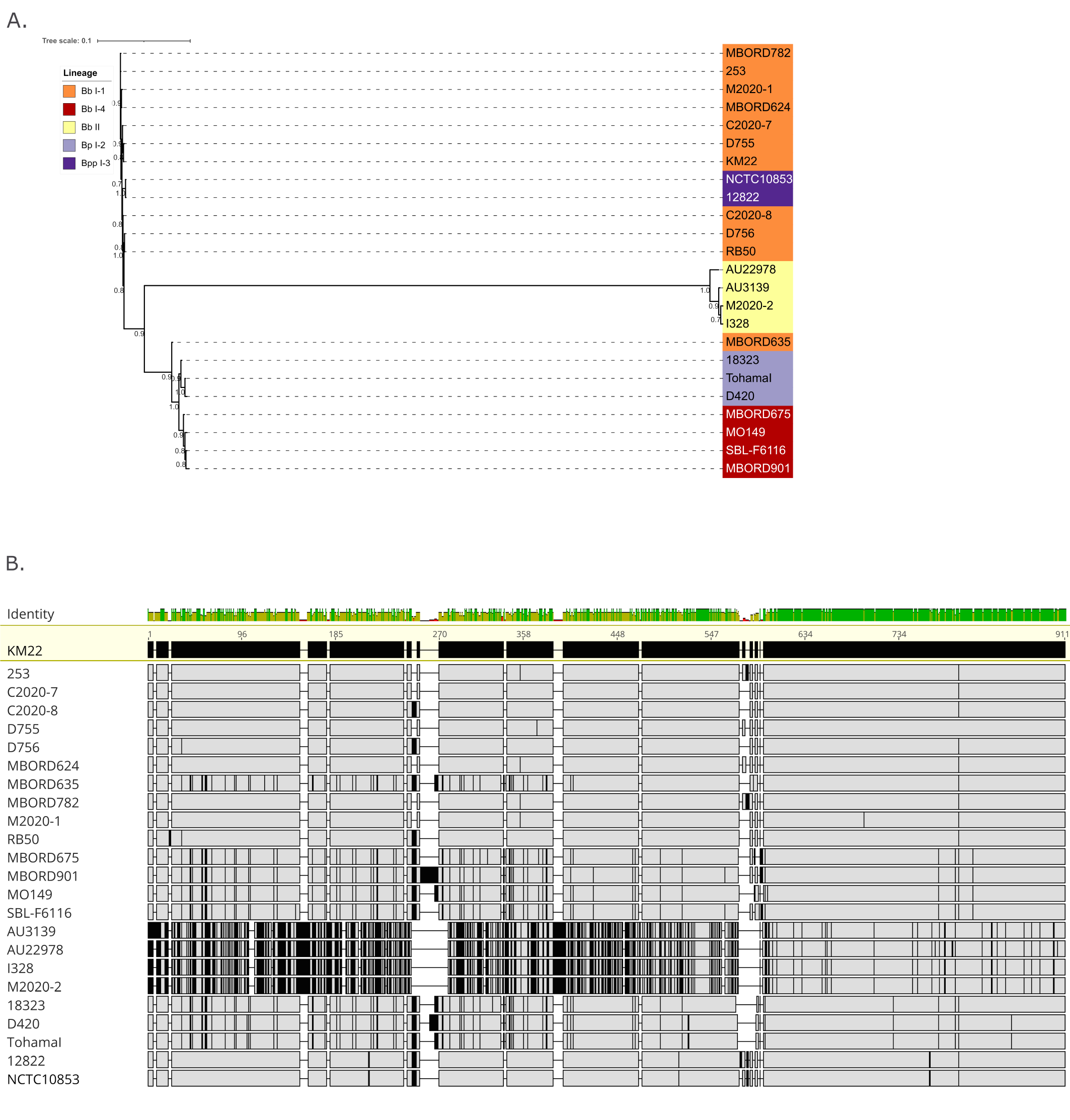

Supplement: SUPPLEMENTARY FIGURE 2 — PRN alignment and phylogenetic tree. (A) Phylogenetic tree inferred from non-redundant PRN AA sequences from analyzed Bordetella isolates containing a full-length prn gene used to build a maximum-likelihood phylogenetic tree with FastTree (v2.11) and iTOL (Letunic and Bork, 2024; Price et al., 2010). Support values are shown on each branch, and isolate names are color coded according to legend shown left of the heatmap. (B) MAFFT alignment of the unique PRN AA sequences from analyzed Bordetella isolates containing a full-length prn gene ordered by lineage. PRN from Bb strain KM22 served as the reference sequence. In the aligned sequences, vertical black lines represent residues that differ from the KM22 FHA reference. The top bar represents mean pairwise identity over all pairs in that column of the alignment; green is 100% identical, yellow is 30 - <100% identical, and red is <30% identical. The height of the bar represents conservation of sequence at that position; a lower height indicates low sequence conservation at that position. [file Image_2.tif]

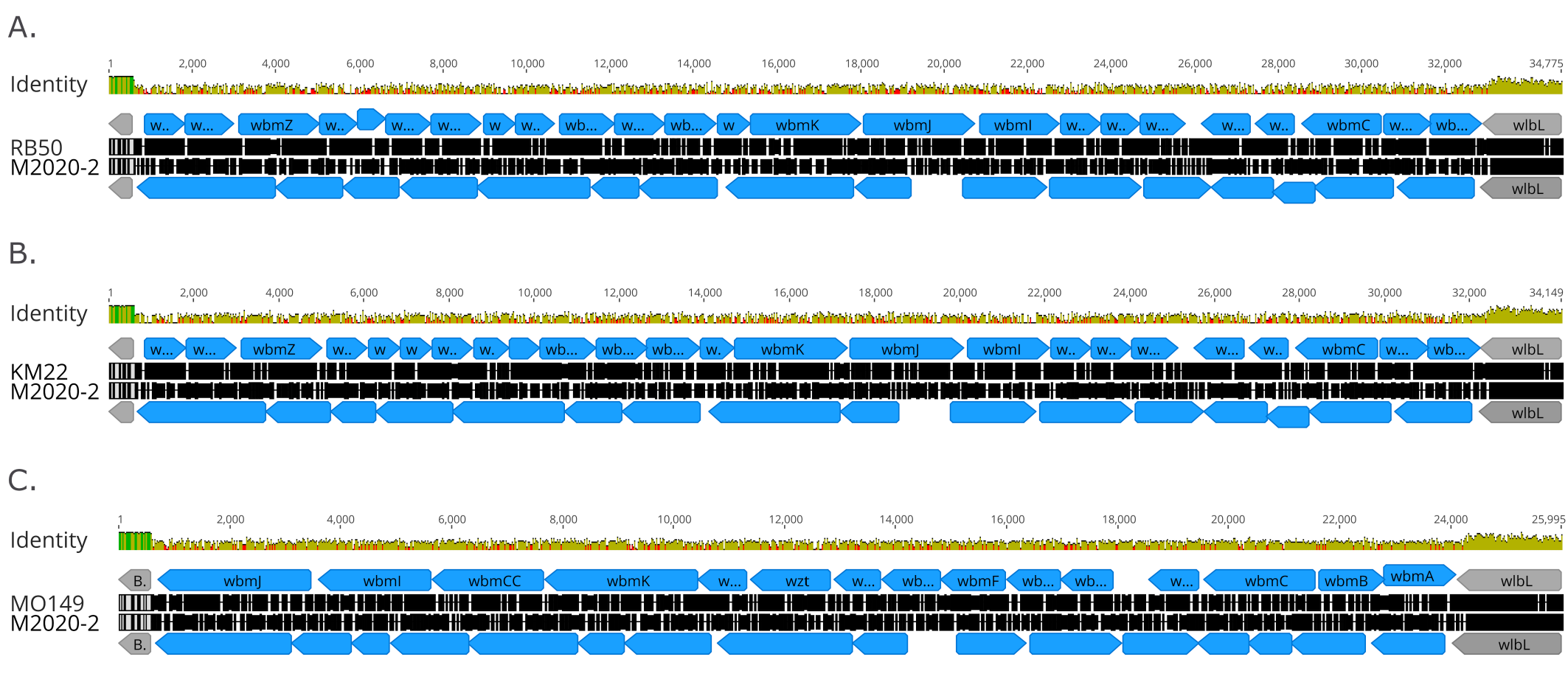

Supplement: SUPPLEMENTARY FIGURE 3 — M2020-2 and M2020-4 do not harbor O-antigen serotype O1, O2, or O3. Alignment of the wbm locus M2020-2 to RB50 serving as reference O-antigen serotype O1 (A), wbm locus M2020-2 to KM22 serving as reference O-antigen serotype O2 (B), and wbm locus M2020-2 to MO149 serving as reference O-antigen serotype O3 (C). M2020-2 and M2020-4 sequences are 100% identical over the region therefore only M2020-2 sequence is used in alignments. Predicted genes within the wbm locus are represented as blue arrows and genes flanking the wbm locus are indicated by grey arrows. Base pair numbers are displayed above black bar at top. In the aligned sequences, vertical black lines represent residues that differ from the reference. The top bar represents mean pairwise identity over all pairs in that column of the alignment; green is 100% identical, yellow is 30 - <100% identical, and red is <30% identical. The height of the bar represents conservation of sequence at that position; a lower height indicates low sequence conservation at that position. [file Image_3.tif]
